# Advancements in Insulin Pumps: A Comprehensive Exploration of Insulin Pump Systems, Technologies, and Future Directions

**DOI:** 10.3390/pharmaceutics16070944

**Published:** 2024-07-15

**Authors:** Mohammad Towhidul Islam Rimon, Md Wasif Hasan, Mohammad Fuad Hassan, Sevki Cesmeci

**Affiliations:** Department of Mechanical Engineering, Georgia Southern University, Statesboro, GA 30458, USA

**Keywords:** Type 1 diabetics (T1D), Type 2 diabetics (T2D), insulin, insulin pump, patch pump, continuous glucose monitoring, micropump

## Abstract

Insulin pumps have transformed the way diabetes is managed by providing a more accurate and individualized method of delivering insulin, in contrast to conventional injection routines. This research explores the progression of insulin pumps, following their advancement from initial ideas to advanced contemporary systems. The report proceeds to categorize insulin pumps according to their delivery systems, specifically differentiating between conventional, patch, and implantable pumps. Every category is thoroughly examined, emphasizing its unique characteristics and capabilities. A comparative examination of commercially available pumps is provided to enhance informed decision making. This section provides a thorough analysis of important specifications among various brands and models. Considered factors include basal rate and bolus dosage capabilities, reservoir size, user interface, and compatibility with other diabetes care tools, such as continuous glucose monitoring (CGM) devices and so on. This review seeks to empower healthcare professionals and patients with the essential information to improve diabetes treatment via individualized pump therapy options. It provides a complete assessment of the development, categorization, and full specification comparisons of insulin pumps.

## 1. Introduction

The landscape of diabetes on a global scale is rapidly evolving, with predictions showing a significant rise in the future. Increasing from the estimated 366 million individuals affected in 2011 to a forecasted 552 million by 2030 highlights the magnitude of this epidemic, emphasizing the urgent need for significant attention and innovative solutions [1]. Type 2 diabetes mellitus (T2DM) comprises most cases, accounting for 85% to 95% globally; type 1 diabetes mellitus (T1DM) cases are increasing alarmingly in selected regions of Europe and the USA, with rates increasing by 2–3% [2]. Consequently, diabetes has become one of the most widespread noncommunicable diseases worldwide. The discovery of insulin stands as one of modern medicine’s greatest achievements, revolutionizing the management of both T1DM and long-standing T2DM [3,4,5]. However, early insulins derived from animal pancreases, such as bovine and porcine, possessed noteworthy challenges, including immunological reactions, erratic absorption, and lipodystrophy [2]. As a result, extensive studies were conducted to overcome the obstacles that slowed down the development of insulin formulations and improve the methods used for insulin purification [4]. During the last couple of decades, fast-acting and long-acting insulin analogs were introduced, indicating a radical turning point of insulin development [2,4]. The Diabetes Control and Complication Trial (DCCT) highlighted the critical role of intensive insulin therapy (IIT) in reducing both microvascular and macrovascular complications among people with type 1 diabetes mellitus (T1DM) [5]. However, the increased risk of hypoglycemia resulting from IIT has lowered the objective of strict glycemic control [6,7]. As a result, the focus has been shifted towards creating insulin formulations that mimic the pancreas’ natural production, with the aim of decreasing the frequency of low blood sugar incidents. Despite subcutaneous injection remaining the preferred method of insulin administration, it has some disadvantages, such as discomfort at the injection site and patients not following through with the treatment [2,4]. Against this backdrop, the landscape of T1DM management has undergone a seismic transformation in recent years. The inception of the first insulin pump in the 1970s introduced a new era of continuous subcutaneous insulin infusion (CSII), laying the groundwork for the pursuit of an artificial pancreatic system (APS) [8,9,10]. This APS, designed to automatically adjust insulin doses based on real-time glucose levels, has been a long-sought goal for scientists and T1DM patients. The evolution of APS has been characterized by successive milestones, from early closed-loop systems which are capable of nocturnal regulation to advanced hybrid closed-loop (HCL) systems facilitating both daytime and nighttime insulin modulation [11,12]. Furthermore, dual hormone HCL systems, integrating glucagon or amylin alongside insulin, have been explored, albeit with varying degrees of success [13]. Recent advancements have culminated in the development of advanced hybrid closed-loop systems (AHCLs), which combine automated basal rate adjustments with correction boluses, yielding superior glycemic outcomes [14,15,16]. Moreover, the emergence of do-it-yourself (DIY) APS systems underscores the community’s proactive engagement in devising innovative solutions, even without clear regulations [17].

While the quest for a fully closed-loop system remains ongoing, contemporary technologies integrating insulin infusion with continuous glucose monitoring (CGM) have garnered widespread adoption among T1DM patients, promising enhanced glycemic control [18,19,20]. Over the decades, researchers and efforts have been made to explore various routes and methods to deliver insulin into the human body. Efforts have also been made to improve the insulin delivery system, including changes and modifications related to design, comfort, automation, and enhanced health monitoring. Insulin pump therapy has emerged as a cornerstone of diabetes management, offering a continuous supply of insulin through subcutaneous infusion with greater flexibility and precision when compared to standard insulin injections. However, the landscape of insulin pump technology is dynamic, with numerous models offering diverse features, delivery methods, and user interfaces. Consequently, conducting a comprehensive comparison of insulin pumps is essential in assisting doctors, healthcare professionals, and patients with diabetes when selecting the most suitable equipment for their specific needs. Additionally, providing an overview of the existing insulin devices on the market, including their clinical and design parameters such as size, weight, basal rate, bolus rate, etc., is necessary. An overview table has been included to present the studies conducted in this regard.

From Table 1, it is evident that some have focused on exploring various routes and challenges associated with insulin delivery, while others have been limited to specific geographical areas. Although a few studies have compared the different insulin pumps available on the market, they have only addressed a limited selection of pumps. There is a need for a more comprehensive study that provides an overview of all insulin pumps, covering their design and clinical parameters on a global scale, which has not been achieved thus far.

Hence, this study aims to fill this gap by not only evaluating insulin pump delivery through various routes, but also providing a comprehensive overview of available insulin pumps worldwide. This overview encompasses their design parameters, clinical conditions, manufacturing details, and more. The study offers a thorough comparative analysis of modern insulin pump technologies, tracking their evolution and classification while assessing their efficacy, characteristics, and user-friendliness. Table 2 represents a guide for the subsequent tables where the comparison is explained. Moreover, this study seeks to identify emerging trends, technological advancements, and potential areas for further research and innovation in the field of insulin pump therapy. By providing evidence-based insights, it aims to empower healthcare professionals and individuals with diabetes to make informed decisions regarding their treatment options.

## 2. Insulin Pump

**Figure 1:**
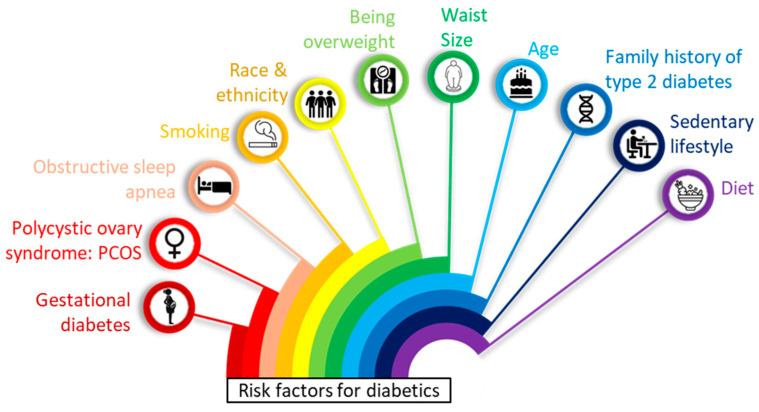
List of potential factors that increase risk diabetics.

Diabetes is an increase in blood glucose levels above normal values. Diabetes occurs because of deficiency of insulin production from the pancreas, the cells cannot use insulin (insulin resistance), or combination of these. Since cells cannot take in the glucose, it builds up in blood resulting in hyperglycemia. The main cause of diabetes varies by type. But no matter what type of diabetes, it can lead to excess sugar in the blood. Too much sugar in the blood can lead to serious health problems. In Figure 1 diabetics risk factors are mentioned. In recent decades, substantial progress in diabetes technologies has resulted in improved glycemic control and reduced complications associated with the microvascular and macrovascular systems [32]. Intensive insulin therapy has evolved from multiple daily injections (MDI) to more advanced technological improvements that have been shown to decrease the chances of hypoglycemia and hyperglycemia, lessen fluctuations in blood sugar levels, and enhance the quality of life for patients [33].

**Figure 2:**
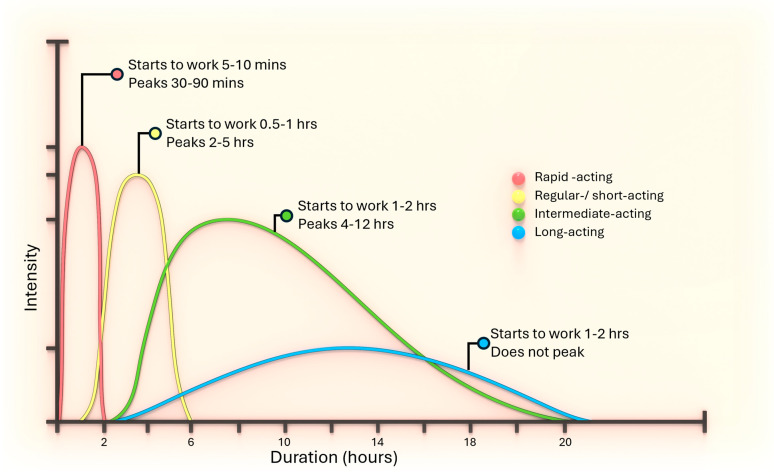
Comparison between insulins type.

In diagnosing diabetic therapy, commonly used insulins are compared in Figure 2. Short-acting insulin has a delayed onset, peaking 2–3 h post-injection, and can cause hypoglycemia due to prolonged action. It forms hexamers post-injection that dissociate into dimers and monomers, thus should be taken 30 min before meals. Rapid-acting insulin, modified to reduce hexamer formation, achieves faster dissolution and absorption, necessitating dose reduction when switching from regular insulin. Examples include insulin lispro, aspart, and glulisine. Intermediate-acting insulin, such as NPH, dissolves slowly due to added protamine or zinc, peaking in 6–14 h with a 10–16 h duration, and can serve as basal insulin when combined with regular or rapid-acting insulins. Zinc insulins, forming crystals, have delayed absorption and prolonged action based on crystal size, and cannot mix with soluble insulin due to excess zinc. Long-acting insulin, like insulin glargine and detemir, provides a steady insulin level with no pronounced peak, making it suitable for maintaining baseline glucose control throughout the day and night. These insulins are designed to be taken once or twice daily to mimic the body’s natural basal insulin secretion.

Insulin pumps, or continuous subcutaneous insulin infusion systems, were the first major step in diabetes technology advancement. These pumps utilized self-monitoring capillary glucose testing to regulate glycemic control and enhance patient satisfaction, surpassing the results obtained with MDI [34].

Over the past 40 years, insulin pump therapy has achieved significant advancements and is now widely used in the contemporary treatment of diabetic patients. Insulin pumps are compact, automated devices that replicate the body’s insulin release more exactly by constantly delivering rapid-acting insulin over the course of 24 h. The system comprises a disposable container for insulin and a disposable set for infusion, which includes a cannula for insertion under the skin and a tubing system that links the insulin container to the cannula. One benefit provided by the pump is the ability to administer a basal infusion of insulin in a quantitative manner. Although basal rates can be established on an hourly basis, they tend to exhibit superior performance for periods of 3–4 h, as insulin takes effect for that duration. Pumps also allow for individualized boluses with meals, preferably depending on the carbohydrate amount of the food, as well as personalized corrective boluses for high glucose corrections. Built-in calculators that propose insulin dosages for meals and corrections while also considering any leftover insulin from earlier boluses and the patient’s insulin action time provide optimal dosing accuracy [35,36].

To keep blood sugar levels stable and avoid ketosis, a gradual and steady injection of insulin, known as basal insulin, is necessary. Although it may differ, the first basal rate is often set at 50% of the total daily insulin demand. This is typically 20–30% lower than when the patient was on MDI treatment. For adult patients, one method to determine the total daily insulin demand is to multiply the body weight in kilograms by 0.5 [37].

The pre-meal bolus insulin maintains euglycemia after meals or corrects above-target glucose levels. The insulin–carbohydrate ratio and carbohydrate intake determine the bolus insulin dosage. Each unit of (100 IU strength) insulin covers a certain quantity of carbohydrate (in grams). The equation for determining the first insulin–carbohydrate ratio is obtained by dividing 450 by the entire daily insulin dosage [37].

Insulin pumps can help patients with a history of hypoglycemia by reducing the frequency of episodes and raising HbA1c levels, which is helpful. Furthermore, for the correct patient group, insulin pump therapy can improve quality of life, provide greater freedom of movement, and reduce hospitalizations and diabetic ketoacidosis (DKA) [38].

Additional considerations include price and insurance coverage. Patients without health insurance may face expenses ranging from $4500 to $6500 for an insulin pump and its associated consumables, depending on the brand. Pump treatment was determined to be less cost-effective than multiple dosage injection (MDI) treatment in an economic analysis for individuals with type 1 diabetes [39].

### 2.1. Evolution of Insulin Pump

**Figure 3:**
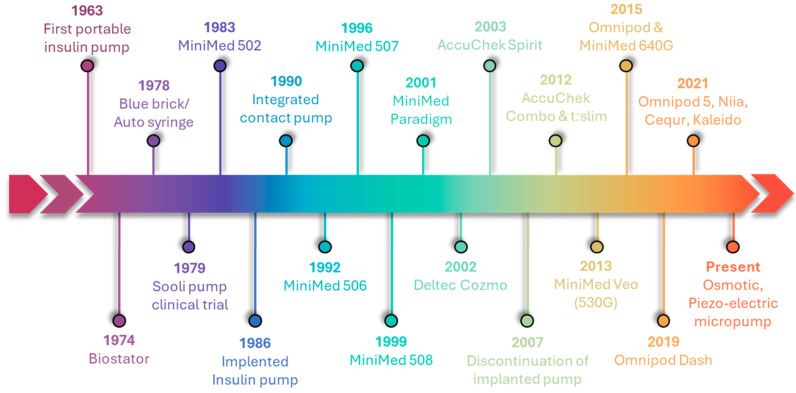
Evolution of insulin pumps.

The development of diabetes technology has been remarkable, starting with the syringe for administering insulin and continuing through insulin pumps, insulin pens, and sensor-augmented pumps The development of these devices has been further accelerated by the introduction of hybrid closed-loop systems, the integration with consumer electronics, and the utilization of cloud-based data systems [40,41]. Insulin pumps have seen tremendous development since their inception over 40 years ago, leading to improvements in size, accuracy, and reliability [42,43,44]. Figure 3 illustrates the chronological development of insulin pumps, progressing from large sizes to smaller micropumps. Dr. Arnold Kadish developed the first portable insulin pump in 1963; nevertheless, its size and technological problems caused it to have limitations [45]. The “Biostator,” an insulin pump including closed-loop intravenous insulin infusion and intravenous continuous glucose monitoring, was created by Dr. Ernst Friedrich Pfeiffer in 1974 at the Miles Laboratory (Elkhart, IN, USA). The system demonstrated the viability of closed-loop glucose management and allowed for subsequent technological advancements, despite its enormous size and complicated operation [46]. In 1978, Dean Kamen brought the first wearable insulin pump—originally called the “blue brick” and then the “autosyringe”—into the world, which marked the beginning of insulin-pump therapy [47]. Only patients waiting for pancreatic transplants were offered these due to their complexity and size. Seoul National University Hospital conducted the initial clinical evaluation of the SOOIL insulin pump in 1979 [48]. The first insulin device available for purchase in the United States appeared in 1979 [49]. MiniMed Technologies founded in 1980, originally known as Pacesstter System, launched their inaugural insulin pump, the MiniMed 502, in 1983. In 1986, MiniMed created the implanted insulin pump for intraperitoneal insulin delivery [50]. During the 1990s, smaller, more compact, portable, and efficient external pumps of the new generation were introduced. These pumps integrate features like bolus calculators, portable diabetics manager, and alerts [51]. In 1992, MiniMed introduced the MiniMed 506, a device equipped with bolus insulin memory. In subsequent years, they enhanced the programmable basal rate and introduced the 507, 507C, and 508 series [52]. In the 2000s, there were notable improvements in the battery life and memory capacity of pumps. Medtronic bought MiniMed in 2001 and then released the MiniMed Paradigm series in subsequent years. In the following years, the paradigm series (511, 512, and 515) experienced enhancements in algorithmic performance and the implementation of a wired continuous glucose monitoring system [52]. In 2002, Smith’s Medical launched Deltec Cozmo, but stopped its production in 2009 for financial issues [52]. In 2003, Medtronic introduced an advanced insulin pump. The system consists of a MiniMed Paradigm 512 insulin pump and a Paradigm Link blood glucose monitor. In modern times, blood glucose (BG) values obtained from the glucometer are communicated electronically and automatically to the insulin pump [53]. In 2006, Accu-Chek Spirit was introduced by Roche Diabetics Care [52]. In the era of the infusion set, the discontinuation of implanted insulin pump devices was announced by Medtronic later in 2007 [54]. The t: slim insulin delivery device from Tandem Diabetics Care and the Accu Chek Combo from Roche were both released in 2012. The introduction of the tubeless insulin pump, Omnipod, was in 2015. After four years, Omnipod Dash was released to consumers [52]. Meditron MiniMed, Omnipod, Tandem, DANA R, Cellnovo, Roche’s Accu-Chek Solo Micropump, and Ypsomed are the insulin pump devices that have received worldwide approval [55].

### 2.2. Candidates for Insulin Pump Therapy

**Figure 4:**
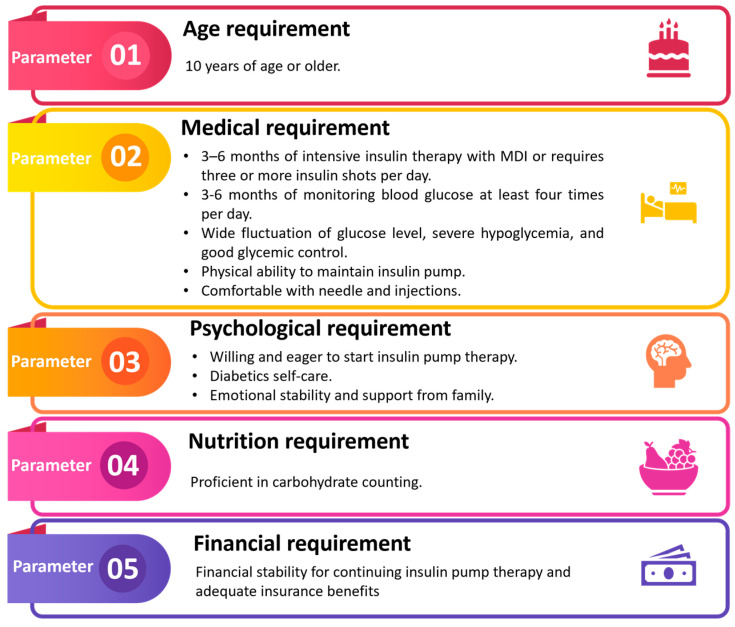
Candidates for pump therapy.

Insulin pumps do not eliminate the need for human interaction or control while dealing with diabetes. It is important for patients to understand that insulin pumps are complex medical devices that still need programming and patient autonomy in managing their condition and insulin dosage. An intelligent, technically competent, and self-motivated patient with a good grasp of the fundamentals of diabetes self-care would be the best candidate to begin insulin pump therapy [56]. Figure 4 illustrates the fundamental prerequisites for a patient who is eligible to utilize the insulin pump. Every patient has to be evaluated by their healthcare professional to see if insulin pump treatment is suitable for them. Healthcare practitioners have a responsibility to inform their patients about the possible advantages and disadvantages, as well as the steps needed to start and keep pace with this type of insulin therapy. The typical age range for individuals prescribed insulin pump treatment is 10 years and above [57].

A patient who shows signs of discomfort or resistance to learning how to utilize the device may not be a good fit. Some patients may benefit more from MDI than pump manipulation due to physical disabilities. When it comes to managing their diabetes and insulin needs, a patient’s intellectual capacity is a key factor. A table outlining the standard procedures for patient selection is provided below.

### 2.3. Insulin Pump Classification

Smart insulin pens, automated insulin administration systems, simpler patch pumps, advanced pen needle technology, and continuous glucose monitoring systems are just a few of the current gadgets that have enhanced care delivery and patient outcomes.

**Figure 5:**
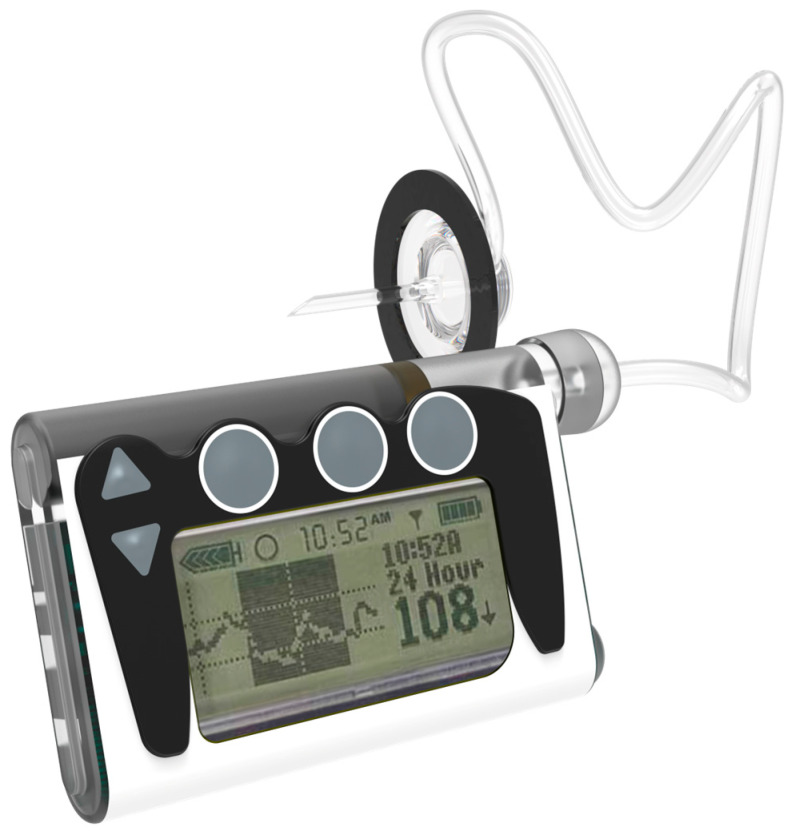
A conventional insulin pump.

**Figure 6:**
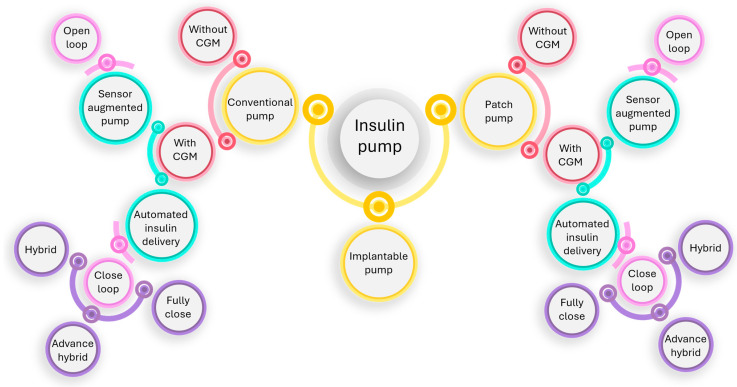
Classification of insulin pump.

An artificial pancreas that incorporates both past and future achievements has been developed by the Juvenile Diabetes Research Foundation, which has been dedicated to the cause for three generations. The original generation of an insulin pump was sensor-augmented pump (Figure 5); the pumps had an insulin shutoff feature that activated when the user did not react to a hypoglycemia warning. The creation of the hybrid closed loop, which regulates the basal insulin rate and needs human input for prandial manual-assist boluses, is a feature of the modern second generation. With the introduction of completely automated multihormone closed loops, the third generation should complete the artificial pancreas principle [58,59]. Figure 6 demonstrates the classification of the three primary types of insulin pumps.

#### 2.3.1. Delivery Mechanism

Insulin pumps had a revolutionary impact on diabetes care through constantly administering insulin using a variety of delivery modes. The three primary ways in which these pumps are categorized are by their delivery mechanism, namely conventional, patch, and implantable. People managing diabetes have a broad variety of options when it comes to insulin administration, with each kind offering unique advantages and considerations.

##### Conventional Pump

**Figure 7:**
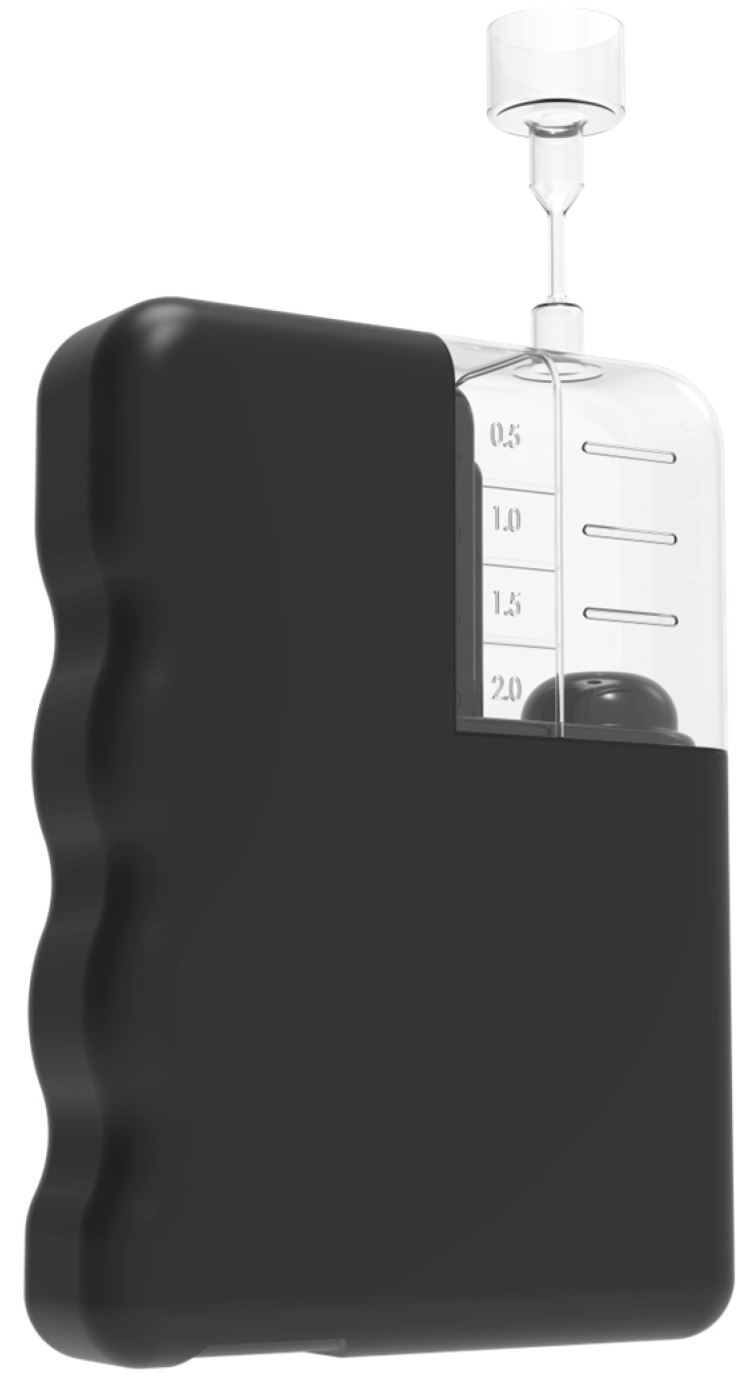
Tandem Mobi, conventional insulin pump.

An insulin pump is a small, digital device that continuously delivers rapid-acting insulin through a small catheter inserted into the subcutaneous tissue and secured in place on the skin with adhesive (referred to as an “infusion set” or “infusion cannula”) [60]. In most insulin pumps, the infusion set connects to the pump by plastic tubing, and insulin infuses from the pump through the tubing to the infusion set cannula and into the subcutaneous tissue. The pump itself, which usually features controls, is free to be tucked into pockets or carried in pump pouches, which can be worn under or outside of clothing. With conventional insulin pumps, the user programs individualized rates for continuous basal insulin delivery to cover all 24 h of the day in increments as small as 0.01 units of insulin/hour. Commonly available examples of tethered pumps are Medtronic 630G, 670G, and Tandem t: slim X2. Tandem Mobi, a representation of contemporary advancement in conventional insulin pump, is illustrated in Figure 7.

##### Patch Pump

To overcome the problems with infusion sets, the so-called patch pumps were developed accordingly. These are insulin pumps devoid of a cannula and are attached directly to the skin by an adhesive [58]. It has several additional advantages, for example, smaller, more discrete, easier to use, and cheaper than conventional insulin pumps. But the downsides are not insignificant. It has to be replaced every 2–3 days; there is also a considerable risk of clogging (mainly by insulin inside the tubing), air bubbles impairing pumping, kinking of the tubing or the Teflon catheter in the subcutaneous tissue, cumbersome handling, and the need for priming [61].

**Figure 8:**
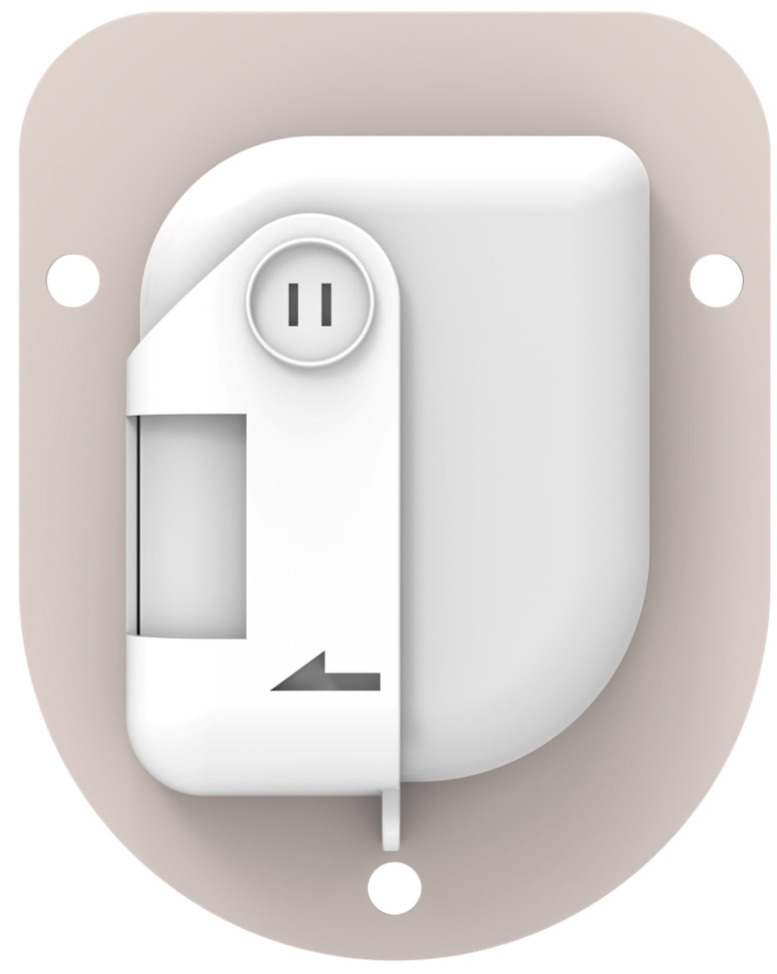
EOpatch, a patch pump developed by a Korean company named EOflow.

The development of flexible insulin reservoirs of different shapes enables the construction of patch pumps that look different from conventional insulin pumps. Pumping insulin requires energy. Thus, appropriate batteries become more of a problem with the smaller electrically driven patch pumps. Most patch pumps require the filling of insulin reservoirs to be completed by the patients themselves; only a few have the option of using prefilled cartridges. Patch pumps come in a variety of shapes, sizes, and technologies. This is also a reflection of varying patient requirements, especially depending on the type of diabetes. There are two tasks in insulin delivery, namely basal rate and bolus [62]. The external and interior schematics of the EOpatch insulin patch pump are shown in Figure 8.

Generally, the different patch pumps can be divided into three categories. The simple forms of PPs are intended for insulin therapy for people with T2D and mainly aim to be easy to handle, easy to carry, small, and disposable. An example of a simple PP is the V-GO (Zealand Pharma; Zealand, Denmark), which delivers a fixed amount of basal insulin over 24 h and has a bolus button that permits up to 36 units of prandial insulin to be delivered in 2-unit increments per day. The V-GO is replaced daily. The Simplicity (CeQur; Luzern, Switzerland) PP holds up to 200 units of bolus insulin that are administered in 2-unit increments, while the CeQur’s PaQ (later PaQ Total) has a reservoir of 330 units for 3 days of use and also allows for different basal rates [63].

Fully equipped pumps can deliver as standard at least variable basal rate(s) and individually controllable amounts of bolus insulin. The first commercially available patch pump termed Omnipod is composed of an integrated infusion set and inserter that communicates wirelessly with an integrated blood glucose meter [64]. The Accu-Chek Solo micropump (Roche Diabetes Care; Mannheim, Germany) is composed of a 90-day reusable pump, a disposable 200-unit insulin reservoir, a disposable pump holder including the cannula, and a remote control. Also, the A6 TouchCare System PP (A6) (Medtrum Technologies, Shanghai, China) has a reusable pump base and a disposable insulin reservoir, including the cannula, in addition to a remote control. The development of the PP Panda (SFC Fluidics, Fayetteville, AR, USA) was supported by the 2017 “Open-Protocol Automated Insulin Delivery Systems Initiative” of the Juvenile Diabetes Research Foundation (JDRF), which aimed to establish an “open-protocol” AID ecosystem. The Sigi PP (AMF Medical; Ecublens, Switzerland) works with readily available prefilled insulin cartridges and is controlled directly from a personal smartphone. The Equil PP (MicroTech Medical; Hangzhou Zhejiang China) has a wireless portable diabetes assistant (PDA). With the GlucoRx Equil (GlucoRx, Guildford, UK), the user can bolus directly from the PP or via a PDA-Bluetooth controller. Medisafe WIT is a removable PP (Terumo; Shibuya, Japan) that, like most other PPs, allows the basal rate and bolus to be adjusted via remote control. The JewelPUMP (Debiotech; Lausanne, Switzerland) also has a separate controller to deliver bolus insulin doses and a reservoir with 450 units of insulin.

For automated insulin delivery patch pumps, according to the FDA, the pump needs to interact with the CGM system and algorithm. An example is the Omnipod 5 system [65]. Via the controlling algorithm, the PP can communicate directly with a Dexcom CGM system and also with a handheld device with the Omnipod 5 App implemented. With this device, the user can start and stop the automated mode, deliver boluses, change settings, and view glucose data and glucose profiles. SFC Fluidics designed its PP Panda to be interoperable with an open protocol that allows a wireless, secure connection to other devices such as the CGM systems or AID algorithms [63].

V-Go (Valeritas) and PAQ (CeQur) are specific simplified patch pump models that are available on the market [66]. In 2013, the second-generation Omnipod, which is smaller and more compact than its predecessor, was launched. This version of the patch pump has advanced features such as ‘‘human factor screens’’ and improvements in both correction and meal boluses for insulin dose calculation [67].

##### Implantable Pump

**Figure 9:**
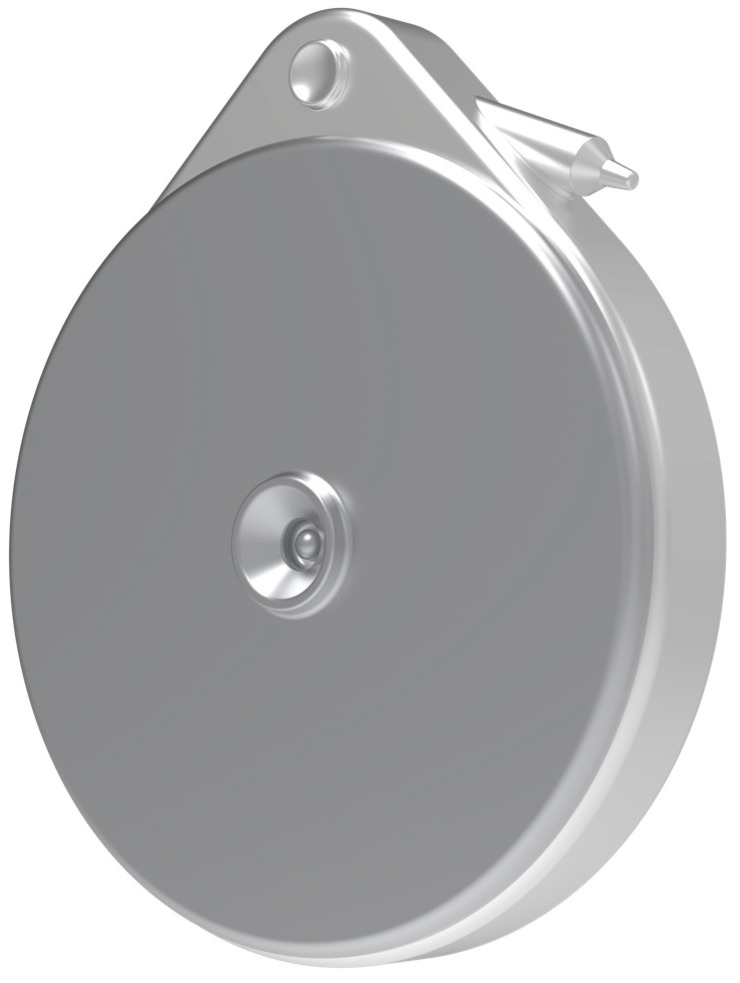
A discontinued implantable pump developed by Medtronic Inc. USA [69].

An implanted insulin pump is a pump which remains at all times into the peritoneal cavity, which has a rich supply of blood vessels and can therefore absorb insulin very efficiently. The use of implanted insulin pumps began enthusiastically a little over 20 years ago. The use of the IP route for type 1 diabetes treatment was made possible by the development of programmable implantable pumps that deliver insulin through an IP catheter. Pilot trials conducted in the 1980s demonstrated the feasibility, efficacy, and safety of this therapeutic approach. Insulin therapy via an implanted pump began in 1989, with its primary development in France. The current implant, the MIP 2007 model (Medtronic-MiniMed, Northridge, CA, USA), underwent improvements to the electronic and battery components of the previous model, has been illustrated in Figure 9 It has been in use since 2000 and has a 7- to 10-year battery life. Insulin delivery options are similar to those of the most up-to-date external pumps and are programmable through a personal pump communicator (PPC). The catheter is inserted into the peritoneal cavity, while the pump itself is implanted in the abdominal wall. In 2007, the MIP 2007 device and Insuplant^®^ 400 IU/mL (Aventis Pharma, Frankfurt, Germany), a semi-synthetic insulin used in implanted pumps, received marketing approval from the French regulatory agency. However, currently, Insuplant 400 IU/mL has been replaced by Insuman Implantable 400 IU/mL (Aventis Pharma), an ordinary recombinant insulin. As with Insuplant, this new insulin has been stabilized to prevent denaturation and precipitation in the implanted pump reservoir [68].

#### 2.3.2. Continuous Glucose Monitoring Integration

Insulin pumps are additionally categorized according to their capacity to integrate with continuous glucose monitoring (CGM) systems, thereby facilitating the more comprehensive monitoring and regulation of blood glucose levels. This classification predominantly comprises two groups, namely automated insulin delivery systems, which incorporate hybrid close-loop pumps, and sensor-augmented pumps.

##### Sensor-Augmented Pump (SAP)

**Figure 10:**
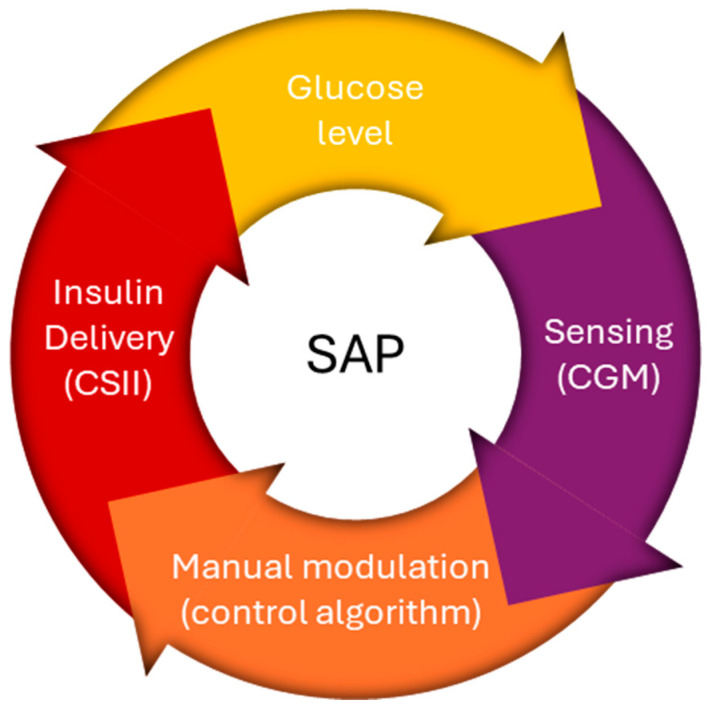
An illustration of the working mechanism of a sensor-augmented pump.

The latest generations of continuous glucose monitors (CGMs) have seen improvements in accuracy and size, benefiting individuals with type 1 diabetes (T1DM) by enhancing glycemic control [70]. When CGM data guide insulin delivery via an insulin pump, it is termed sensor-augmented pump (SAP) therapy or open-loop technology. The SAP platform integrates two independent technologies into a single system. The functioning mechanism of the sensor-augmented pump is illustrated in Figure 10. Utilizing a glucose sensor for continuous, frequent glucose measurements (CGM) has shown significant advantages, particularly in comparison to other treatment methods. This sensor-based approach not only provides a crucial element for the potential transition from open-loop to closed-loop systems, but also transforms continuous subcutaneous insulin infusion (CSII) into a distinct therapy [58,71].

There are currently two generations of SAPs on the market as follows: in the first, the insulin-dosing software operates independently of the CGM values so that the user has to make basal rate adjustments manually; in the second, the insulin-dosing software and the CGM values are coupled, which allows for the automated suspension of basal insulin delivery in response to a predicted or detected low glucose level [71].

Medtronic pioneered integrated diabetes management with the MiniMed Paradigm REAL-Time system in 2006, blending an insulin pump with continuous glucose monitoring (CGM) [64]. In 2009, Medtronic launched the MiniMed Veo System, with a Low Glucose Suspend feature that automatically halts insulin delivery when sensor glucose levels hit a preset low threshold. Insulin delivery resumes automatically after 120 min irrespective of the glucose level, but can be restarted beforehand by the user [71]. The 2015 Medtronic MiniMed 640G further advanced this feature by automatically pausing insulin based on predicted and current glucose readings within 70 mg/dL that were above the lower limit and predicted to be approaching the low limit threshold in 30 min [58,71].

##### Automated Insulin Delivery (AID)

The goal of modern diabetes technology is the development of an automated insulin delivery system that attempts to replace the lack of insulin in diabetic patients in a manner that mimics endogenous insulin secretion. The AID system does not require any input from users. The development of AID, sometimes called the artificial pancreas, began with clinical experiments in the 1960s. Progress in hardware and artificial intelligence empowered further development, leading to the first hybrid closed-loop AID in 2018 [72].

**Figure 11:**
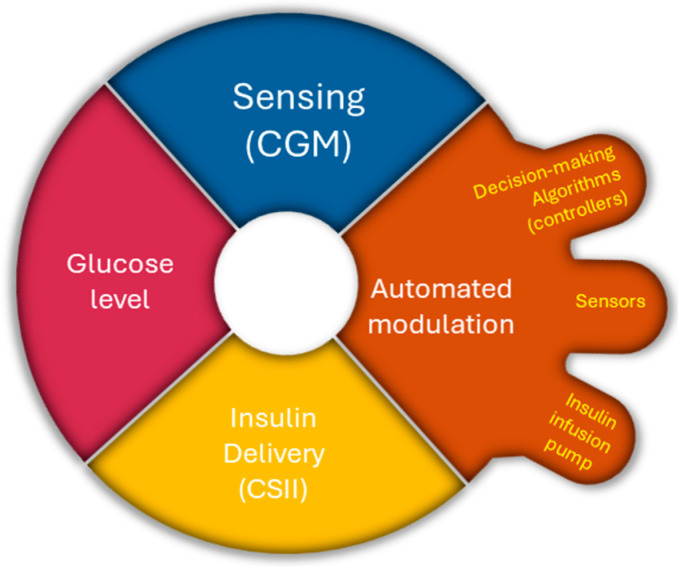
An illustration of the working mechanism of an AID pump.

AIDs have three main components as follows: sensors, decision-making algorithms (controllers), and insulin infusion pumps. Three different control algorithms with significantly different approaches are used for AIDs, namely proportional-integral-derivative (PID) control, model predictive control (MPC), and fuzzy-logic knowledge-based systems, which integrate sensor data and automate insulin delivery [73]. Ideally, automated insulin delivery algorithms take into consideration the current glucose value and should predict glucose values over a given period [74]. Figure 11 illustrates the operational mechanism of the AID pump.

##### Hybrid Closed-Loop (HCL)

Hybrid closed loops, which require limited user input in the form of carbohydrate counting to calculate and deliver prandial boluses, are a more feasible option [75].

**Figure 12:**
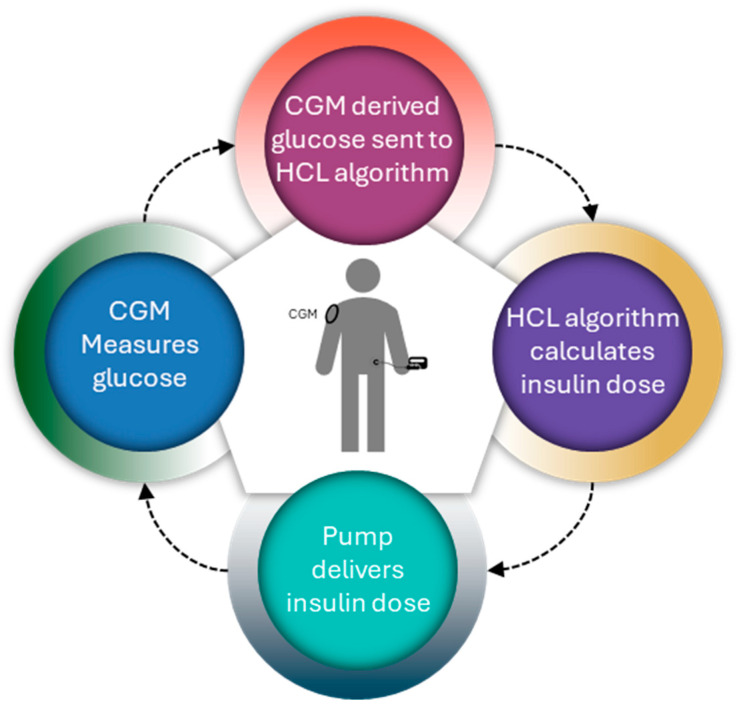
An illustration of the working mechanism of an HCL pump.

In 2017, the first hybrid closed-loop system, the MiniMed 670G insulin pump with a Guardian 3 sensor, was approved by the FDA [54]. When in auto mode, it functions as a hybrid closed-loop system that automatically controls basal insulin delivery every 5 min based on the CGM values to maintain BG levels close to the specific target [76]. The functioning diagram of the hybrid closed-loop pump system appears in Figure 12.

##### Advanced Hybrid Closed-Loop (AHCL)

Recently, advanced hybrid closed-loop systems have been introduced, with the development of more sophisticated algorithms that deliver and modulate basal insulin and allow automatic corrective boluses to be delivered in case of hyperglycemia [77]. Advanced hybrid closed-loop systems offer improved glucose control and represent the most advanced form of insulin delivery currently available for diabetic patients; however, the extensive manual input required by the user in the form of carbohydrate counting and bolus delivery, alarm fatigue, fear of hypoglycemia, and diabetes-related psychological distress has highlighted the need for further research into newer technologies [58]. The FDA approved the advanced hybrid close-loop Medtronic MiniMed 780G system for people with type 1 diabetes aged 7 years and older.

##### Fully Closed-Loop

**Figure 13:**
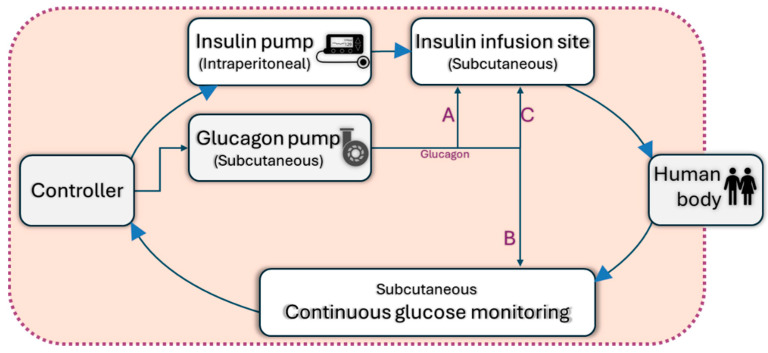
Illustration of a subcutaneous (SC) artificial pancreas with possible uses of glucagon to achieve a fully closed-loop system.

Over time, these systems evolved and incorporated continuous insulin secretion systems with a real-time CGM, controlled by complex mathematical algorithms that determine the amount of insulin delivered subcutaneously based on the glucose value recorded by CGM, termed closed-loop systems [78]. A schematic for a fully closed-loop pump has been shown in Figure 13. Nonetheless, “fully closed-loop” automated insulin delivery system development remains an arduous goal and is hindered by the non-linear relationship between subcutaneous insulin administration and postprandial glucose due to unforeseeable trends in glucose absorption following meals characterized by post-prandial hyperglycemic excursions and tardive post-prandial hypoglycemia due to the delay in the insulin action of currently available rapid-acting insulin analogues [58]. Currently, the only commercially available fully closed-loop system is the STG-55 (Nikkiso, Tokyo, Japan) and its predecessor, the STG-22 [79].

## 3. Comparative Study of Insulin Pump

### 3.1. Criteria to Meet

Insulin pumps undergo evaluation based on many essential factors that are vital for efficient diabetes treatment. This evaluation ensures that the pumps fulfill users’ requirements and effectively tackle significant challenges, all while maintaining a user-friendly interface.

#### 3.1.1. Energy Efficiency and Varied Requirements

Insulin pumps are becoming smaller in size due to the requirement for patients to always carry them. Nevertheless, the quality of being small presents difficulties, and one of them is the duration of the battery. The pump must dispense insulin from the reservoir, and the actuation system of the pump must be energy-efficient. The minimum efficiency rate is 35 units per hour, with an accuracy of 0.05 units [80]. Most insulin pumps available for purchase are powered by either batteries or mechanical mechanisms. Nevertheless, other conceptual models have been presented that utilize piezoelectric, electromagnetic, and saline solution technologies. An implanted micropump based on a conducting polymer called polypyrrole (PPy) has been suggested. This micropump uses a power of less than 2 mW and operates at a voltage below 1.5 V [81].

#### 3.1.2. Innovative Technological Solutions

The continuous glucose monitoring system and insulin pumps are advancing via the integration of cutting-edge technology and the prioritization of patient adaptability. The Dexcom G7 is the most recent iteration of the Dexcom G6, boasting improved accuracy compared to its previous model. The all-in-one sensor and transmitter have a warm-up time of 30 min, which is shorter than the G6’s need of two hours. The G7 incorporates a Quiet Mode and Delayed 1st Alert functionality, in addition to the Urgent Low Alert feature, which is also included in the G6. The Libre 3 is a device designed for those aged 4 years and older, which continuously monitors blood sugar levels without the need for finger sticks. It provides alerts for both high and low levels of glucose. The Eversense 3 system consists of an implanted sensor with a longer lifespan of 180 days compared to the prior two models [82].

The Control-IQ technology utilized in the t: slim X2 insulin pump accurately forecasts glucose levels with a 30 min lead time and automatically modifies basal rates to mitigate the risk of hyper and hypoglycemia. The MiniMed 780G is a hybrid closed-loop device that administers insulin at five-minute intervals. The device is equipped with Medtronic SmartGuard technology and Guardian Sensor 4. The Omnipod is the initial tubeless pump that has received approval from the FDA. The gadget utilizes the Dexcom G6 Continuous Glucose Monitoring (CGM) system and may be operated via either a smartphone or a separate controller device. The Beta Bionics iLet entirely automates the administration of insulin dosages. The selection includes three types of insulin as follows: Novolog, Humalog, and Fiasp Pumpcart [82].

#### 3.1.3. Addressing Practical Challenges

Practical challenges that must be resolved to ensure the highest level of user experience and safety are in addition to the primary criteria that insulin pumps must fulfill. Insulin pumps must not only fulfill essential requirements, but also effectively tackle practical obstacles to provide an ideal user experience and assure safety. Some users may experience a little discomfort during insertion, emphasizing the importance of enhancing insertion procedures and providing better user assistance. To avoid issues, it is necessary to carefully prepare and regularly examine the places where medical devices are inserted in order to prevent skin problems, including irritation and infections [83]. The management of low blood sugar (hypoglycemia) and high blood sugar (hyperglycemia) is still a concern. This requires the application of advanced algorithms and educating users to reduce the dangers associated with these conditions. Diabetic ketoacidosis, a serious consequence, highlights the significance of diligent glucose monitoring and prompt management [83]. Some pumps could have mechanical errors, for instance, tunneling or the blockage of the infusion pipe. Insulin leaking from the tubing or reservoir might result in elevated blood glucose levels [84]. If there is a suspicion of a clog, leak, or issue linked to infusion, it is necessary to replace or fix the complete infusion set. Insulin pumps can provide users with a safer and more comfortable alternative for managing diabetes through effectively tackling these practical issues.

### 3.2. Specification Comparison

Insulin pump specifications include a variety of characteristics such as different infusion set options, methods of delivering insulin, calculators for determining bolus doses, capabilities for connecting to other devices, and designs for the user interface. These parameters across different insulin pump models provide valuable information to the patients about the features of each pump and make an informed decision about which one suits their lifestyle and treatment objectives.

#### 3.2.1. Manufacturer and Releasing Year

Insulin pump manufacturers provide a wide variety of pump types, each with unique characteristics and specifications designed to fulfill the different requirements of individual diabetics. Medtronic, Tandem Diabetes Care, Insulet Corporation, and Roche Diabetes Care are some of the renowned manufacturers. Over time, some pump models have been terminated as companies modify their product offers. In 2017, Animas Corporation, a subsidiary of Johnson & Johnson Diabetes Care Companies, declared the termination of its Animas Vibe and OneTouch Ping insulin pumps, signifying its departure from the insulin pump industry [85]. In addition, corporations frequently discontinue outdated models in order to promote newer, enhanced variants. For example, the Omnipod system has undergone modifications and developments over time, with newer versions, Omnipod 5, replacing older ones, Omnipod, to improve user experience and functionality [86]. Table 3 contains a comprehensive list of the manufacturers of both patchable and non-patchable pumps, as well as the year of their release.

#### 3.2.2. FDA Approval and Origin

The Food and Drug Administration (FDA) in the United States is responsible for regulating and granting permission for medical equipment, such as insulin pumps. Manufacturers are required to establish the safety, efficacy, and quality of their pumps through conducting clinical trials and thorough testing in order to receive clearance from the FDA.

Insulin pumps are produced by enterprises from various countries worldwide. Several leading manufacturers, like Medtronic, Tandem Diabetes Care, Insulet Corporation, and Roche Diabetes Care, have headquarters or production facilities located in several countries, such as the United States, Ireland, Switzerland, and other nations. Table 4 provides a comprehensive list of FDA-approved or certified patchable and non-patchable pumps, together with information on their availability in different regions.

Apart from the pumps mentioned above, several are still under development or have requested FDA clearance. For example, SFC Fluidics Inc., located in the United States, has been in the patch pump industry since 2003, and, in 2020, SFC Fluidics Inc. acquired FDA breakthrough device classification [165]. Unilife, a firm located in the United States, has claimed to possess the world’s first instant patch pump. However, it has not yet obtained any certification [166]. The Sigi patch pump, which was initially developed by AMF Medical SA in Switzerland, is currently being developed by Tandem, USA [167]. Another Swiss company, Debiotech, demonstrated JewelPump in 2010, but has not received FDA or EU clearance [66]. The Dibkit insulin pump, developed by the IA Collaborative and the Osmotic WBI and created by Subcuject, has not yet obtained FDA certification.

#### 3.2.3. Pumping Mechanism

**Figure 14:**
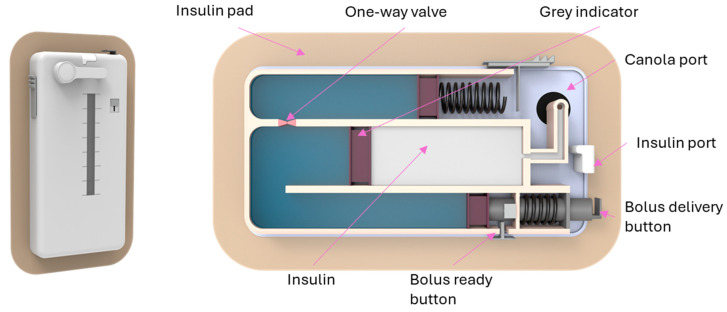
V-Go pump mechanism.

A traditional insulin pump uses a stepper motor to actuate a plunger which delivers insulin to the user. The device is powered by a battery and controlled with a simple PCB and microcontroller. Users are able to enter simple commands via buttons to control the insulin their pump delivers. V-Go has been available on the market for several years, and it was created mainly for individuals with type 2 diabetes. As shown in Figure 14, the drive mechanism consists of a lead screw (1), a plunger attached to a lead-screw follower (2), and a compression spring (3). If the rotation of the follower is restricted, the restoring force from the compressed spring will drive the plunger forwards, causing the lead screw to rotate. The insulin dosage can be controlled by limiting the rotation of the lead screw using a clockwork escapement mechanism (5), so that the periodic release of an escapement gear delivers a set dose of insulin.

The escapement is connected to the lead screw through a 50:1 gearbox (4), so that one ‘tick’ of the escapement delivers a 0.1-unit (0.001 mL) increment of U-100 insulin. A solenoid will be used to actuate the escapement so that the dosage can be controlled via Bluetooth [168]. This tiny insulin-filled device weighs less than 2 ounces and is inches in size. It is designed for single-day use. It offers 20, 30, or 40 units/day as its three different basal rate models. The patient only needs to press a delivery button and a lock release to release two units of insulin during an insulin bolus. Prior to every delivery of two units, the lock release must be pressed. With 18 of these 2-unit dosages permitted daily by the device, a maximum daily dose of 76 units (40 basal + 36 boluses) is possible. V-Go is compatible with U100 Aspart and U100 Lispro insulins; however, it appears that V-Go is not approved for U200 Lispro, which has a 152-unit daily dose total. Notably, V-Go is only offered as a pen form and does not support this more concentrated type of insulin. The V-Go device is covered by several insurance policies, including several Medicare Part D plans.

The Solo Micropump Insulin Delivery System (Medingo US, Inc., Tampa, FL, USA), which was later acquired by Roche Diagnostics, consists of a basal bolus micropump, wireless remote controller, and cradle with a built-in cannula. The 2 mL insulin reservoir, which attaches to the pump, must be replaced at least every 2 days when insulin Lispro or insulin Glulisine is used, and at least every 3 days when insulin Aspart is used. The pump itself should be replaced every 90 days [169].

**Figure 15:**
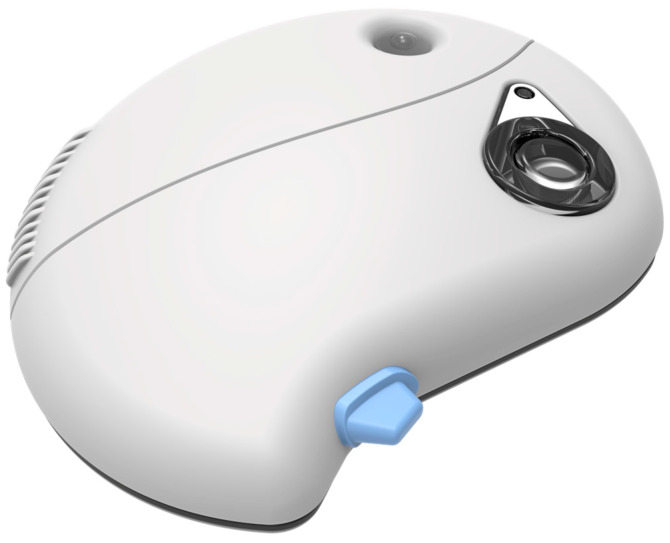
The PAQ by CeQur.

The CE-certified PAQ is available in Europe and consists of a small, reusable electronic portion and a disposable insulin-filled component (Figure 15). This novel device can hold up to 330 units of U100 insulin and runs for three days. There are multiple types available, with varying basal rates spanning from 16 to 60 units every 24 h cycle. Users can click a button meant to avoid unintentional activation to administer a bolus, which delivers two units of insulin. The PAQ has a dose count card and alerts to remind users to change their units even if it does not have an insulin meter. The device, which is about 3 inches wide, works by stretching a balloon that is the device’s power source with insulin being introduced through a port on the underside. There is a flow restrictor system in place to control the basal rate.

**Figure 16:**
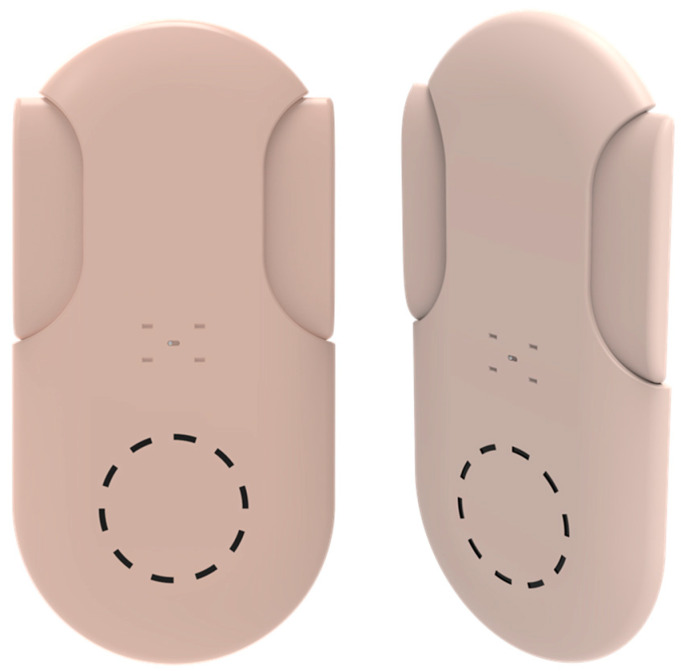
CeQur simplicity by CeQur.

CeQur simplicity by CeQur is another patchable insulin pump for type 1 diabetic patients. This device, initially developed by Calibra, has FDA clearance, but has never been marketed (Figure 16). It is designed as an insulin pen replacement, providing boluses but no basal insulin. The 3-day device can hold up to 200 units of U100 insulin. Pressing the buttons on both sides of the device delivers 1 or 2 units of insulin, depending on the model. There is no insulin delivery counter. The device will lock up if clogged. After the insertion needle quickly retracts, it leaves a small, flexible cannula in your subcutaneous tissue (body fat) that will deliver your insulin. To actually deliver a dose of insulin when you are eating or correcting high blood sugar, you simply squeeze the buttons on both sides of the patch.

**Figure 17:**
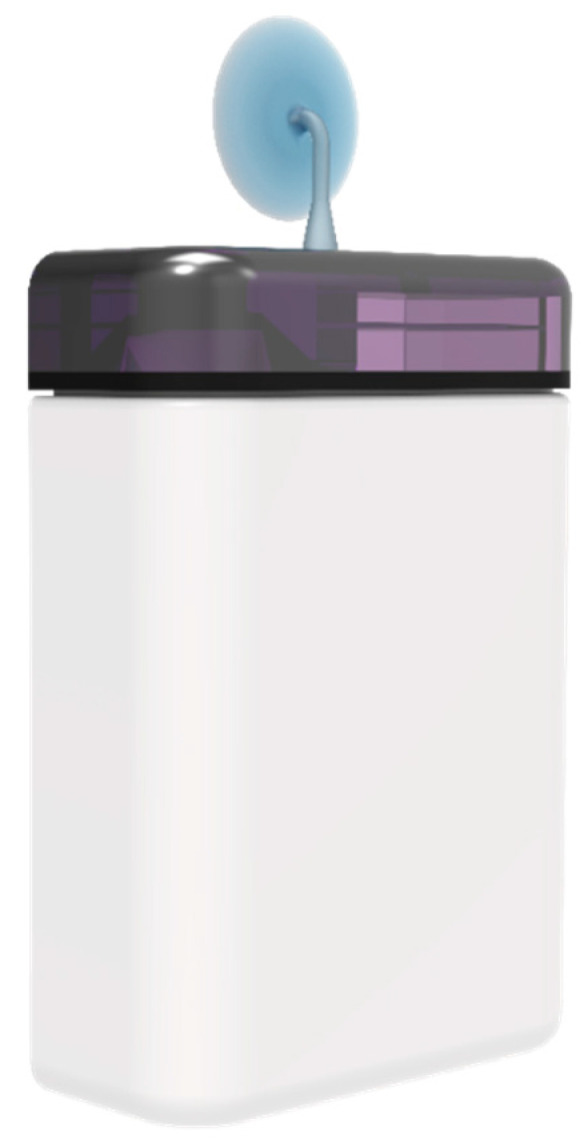
Cellnovo patch pump.

The Cellnovo system, available in Europe, includes a compact patch pump measuring about 2 × 1.5 × 0.6 inches, which is adhered to the skin via a short tubing leading to a cannula (Figure 17). This pump employs a 150-unit disposable cartridge, granting some users a 3-day supply, although many can use it for only 2 days. The pump itself is reusable and detachable for activities like showering or swimming, controlled via a “smartphone-like” device.

**Figure 18:**
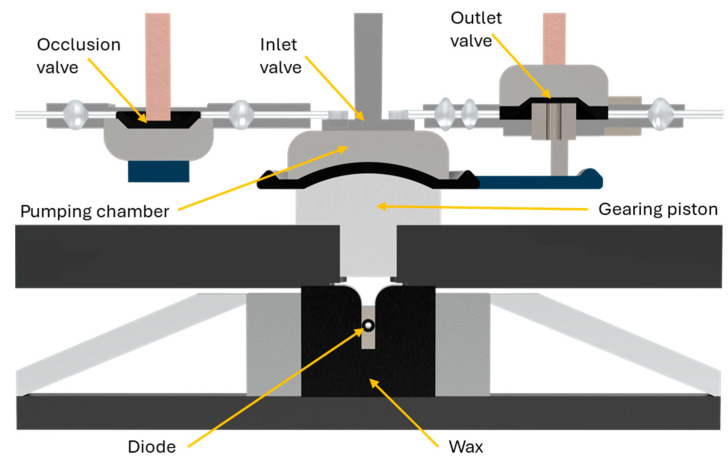
The working mechanism of Cellnovo.

The system offers a range of basal rates from 0.05 to 5 units per hour and boluses from 0.05 to 30 units. It utilizes a unique “Wax Engine” for insulin delivery, which is slower (1 unit per minute) compared to other pumps, necessitating a longer interval between bolusing and eating. Immediate, extended, dual, and multi-phase bolus patterns are available. To detect occlusions, approximately 1 unit of insulin is required, equivalent to about 60–90 min at a basal rate of 0.8 units per hour. The “Wax Engine” functions by heating a wax block, causing it to expand and push a plunger, ultimately delivering insulin (Figure 18). When the wax cools, it contracts, retracting the plunger and changing the valve configuration for insulin delivery.

**Figure 19:**
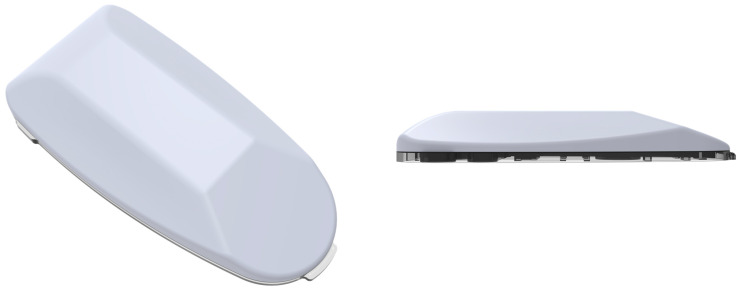
The JewelPump by Debiotech.

Debiotech showcased the JewelPump at the 2010 American Diabetes Association Scientific Sessions. Although it is still promoted on its website and currently undergoing clinical trials, JewelPump lacks FDA and EU clearance. Despite this, its unique features are worth discussing (refer to Figure 19). Utilizing a piezoelectric crystal, the pump, measuring 2.6 × 1.6 × 0.6 inches, can hold 500 units of insulin. The disposable section (depicted on the left in Figure) comprises a pump with a reservoir and a detachable needle. Piezoelectric crystals, commonly used in electronics as speakers, bend when a current passes through them. Debiotech’s innovative pump employs this bending motion to propel insulin. Unlike early piezoelectric pumps, which suffered from inconsistent pumping volumes, Debiotech’s design appears accurate enough to eliminate the need for additional fluid measuring devices. 

**Figure 20:**
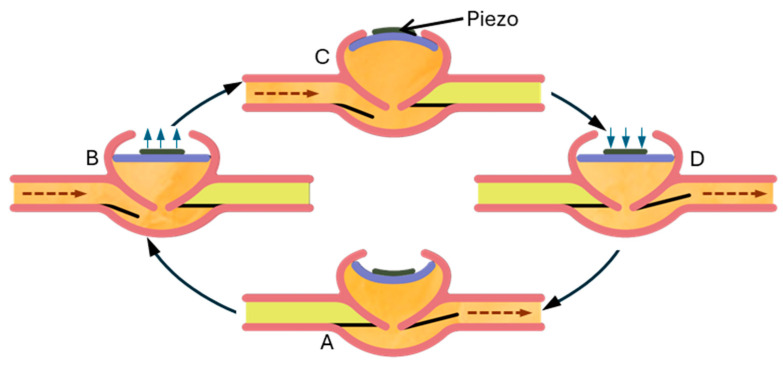
Working mechanism of the piezoelectric insulin pump (JewelPump).

In Figure 20, a negative current causes the piezo to bend downward (A), displacing fluid in the pumping chamber. When the current stops, the piezo flattens (B), drawing insulin into the chamber. With a positive current, the piezo bends upward (C), drawing more insulin in. When the current ceases again, the piezo returns flat (D), expelling insulin through the outlet valve while the inlet valve closes. Reapplying a negative current discharge the remaining insulin. The lower image displays the full size of the crystal and valves.

**Figure 21:**
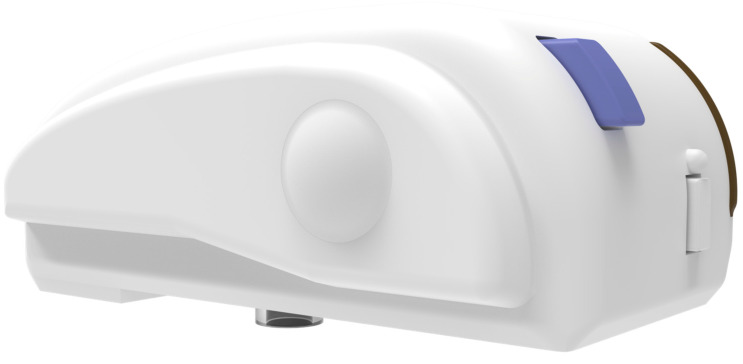
Accu Check by Roche Diabetes Care GmbH.

Recently, the tubeless Accu-Chek^®^ Solo micropump system (Roche Diabetes Care GmbH, Vienna, Austria) was CE-marked and is currently marketed in selected European countries (Figure 21). This is a compact patch pump measuring about 63 × 39 × 14 mm. The pump holder features an adhesive patch and, apart from carrying the micropump, it also fixes the soft cannula in place. This pump employs a 200-unit disposable cartridge, granting some users a 3-day supply, although many can use it for only 2 days. The weight of the pump is less than 29 g, and it is compatible with insulin like Humalog, NovoLog, NovoRapid, Fiasp, Apidra, and Insuman Infusa. The bolus amount is 0.2–50 U. The micropump comprises three key components as follows: a reusable pump base, a disposable insulin reservoir, and a pump holder with an adhesive patch. The pump base houses all electronic components, including the drive, buzzer, and quick bolus buttons. The pump holder attaches the micropump to the body, securing both the pump and the soft cannula. Users can initiate insulin bolus deliveries through the diabetes manager or the pump’s quick bolus buttons, providing flexibility in administration methods.

The innovative MEDISAFE WITH™ (MW) insulin patch pump, developed by Terumo Corporation in Tokyo, Japan, comprises separate pump and controller units. The controller and display features are integrated into a wireless remote control, equipped with an LCD touch panel for users to adjust insulin delivery settings and input operational commands, allowing the wireless control of the pump.

**Figure 22:**
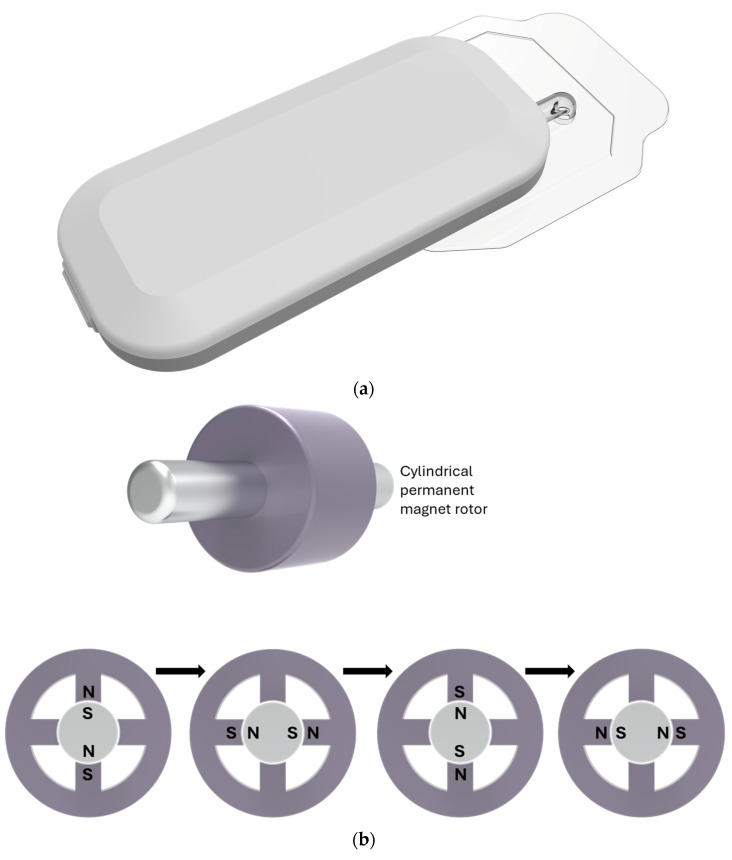
Medisafe with™ (MW), (**a**) pump components; (**b**) working mechanism.

All pump components, including the pump body, tubing, cannula, and cartridge, are combined into a single unit which is designed to adhere directly to the user’s skin. The MW utilizes a small stepping motor called Icuradrive™ for its operation, as demonstrated in Figure 22. In a simplified example, the motor consists of a rotor with two magnetic poles and four electromagnets arranged around it. Pulsed currents, initiated by the remote control, interact with these electromagnets, generating attractive and repulsive forces that drive the rotation of the motor. In this instance, a single pulse current corresponds to a quarter of a revolution. The motor’s rotation is then transferred to gears, which propel a piston inside the cartridge to initiate insulin delivery. The pulse drive method ensures that the motor does not rotate independently, even in the event of hardware failures, such as an electric circuit malfunction. Furthermore, a rotation sensor, directly connected to the motor axis, continuously monitors the motor’s rotation status for added safety.

**Figure 23:**
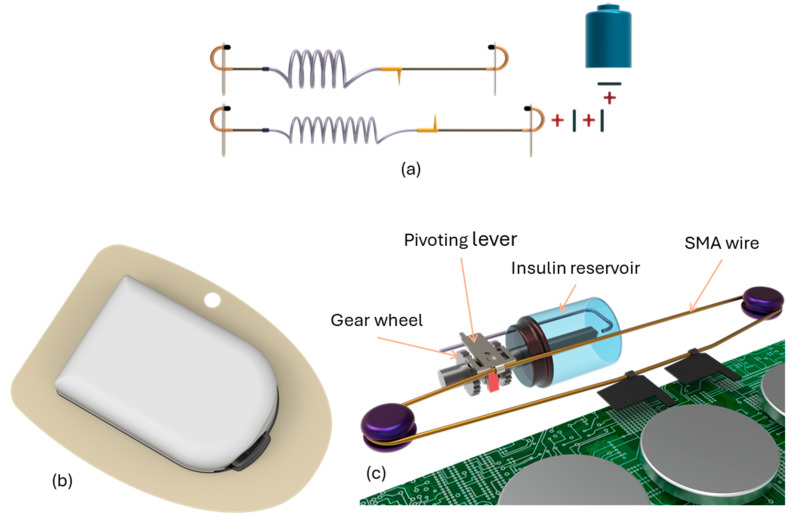
(**a**) Shape memory alloy technology demonstration; (**b**) Omnipod Insulin pump by Insulet; (**c**) pumping mechanism.

There is limited information available regarding the remaining pumps. The SFC Fluidics pump utilizes electrically induced changes in osmolality to drive water osmotically, pushing on a pumping chamber to deliver insulin. An electrical current in a chamber transforms large molecules into smaller, more osmotic molecules, creating an osmotic solvent flow from the left chamber through a semipermeable inter-chamber membrane to the right chamber. This action causes the chamber to expand its impermeable flexible membrane into the pumping chamber facilitating insulin delivery. A second pumping chamber can be added on the left for a different hormone. Becton Dickinson has previewed a new wearable autoinjector capable of delivering various medications, including insulin. While specific details are limited, the pump will come with its own cartridge, support both basal and bolus dosing, and is expected to become available in 2019. EOFlow has introduced its EOPatch, a patch pump similar in appearance to the Omnipod (Figure 23) but slightly smaller. Other pumps, such as NIA Essentials and the P6 Easy Patch Disposable Pump, are currently in their trial phases and are available in the market. As technology continues to advance, the future of patch pumps holds the promise of even smaller, more efficient, and user-friendly devices. With ongoing research and development, these pumps are likely to become increasingly sophisticated, addressing not only the physiological aspects of insulin delivery, but also the practical concerns of patients, ultimately improving diabetes management and quality of life.

#### 3.2.4. Technological Advancement

**Figure 24:**
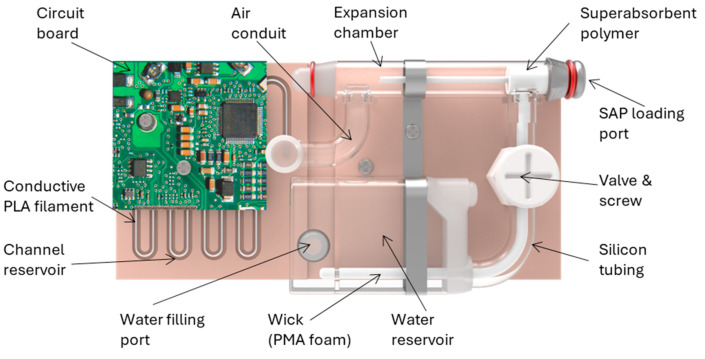
A 3D model of a self-powered insulin pump.

Shao et al. have developed a novel self-powered insulin patch pump utilizing sodium polyacrylate which is a superabsorbent polymer (SAP, as an innovative battery alternative [170]. This proposed patch pump (depicted in Figure 24) comprises a channel reservoir, an expansion chamber, and a water reservoir. The channel reservoir, constructed from laser-cut acrylic sheet and lined with conductive polylactic acid filament, holds 350 mL of infusate, featuring a hydrophobic coating. The cylindrical expansion chamber contains SAP (ID = 4 mm, OD = 8 mm, length = 35 mm), connected to the 800 mL water reservoir via a wick and silicone tubing, incorporating an air-permeable membrane.

Operation commences by opening the valve, enabling deionized water to hydrate the SAP, and creating pressure that initiates infusion from the outlet. Time-lapsed images display superabsorbent polymer expansion at 0, 10, and 20 min. The patch pump consistently infuses over 250 mL of 0.1 M phosphate-buffered saline within 20 min using 50 mg of polymer. By adjusting the valve, infusion rates of up to 15.8 mL min^−1^ and 6.1 mL min^−1^ were achieved during rapid and controlled infusion phases. Fully opening the valve increased the rates to 49.1 mL min^−1^ and 9.3 mL min^−1^, allowing for the basal and bolus infusion rates of approximately 10 mL h^−1^ (1 U h^−1^) and 100 mL (10 U) in around 11 min for effective glycemic control. The generated pressure of around 0.7 psi exceeds the adult’s maximum peripheral venous pressure of 0.6 psi, ensuring adequate infusion.

This innovative approach not only provides an eco-friendly solution, but also prevents over 100,000 used button cell batteries from entering medical waste streams and landfills daily, contributing significantly to environmental sustainability.

#### 3.2.5. Pumping Power

Insulin devices conventionally depend on batteries for their operational energy. Depending on the pump model, these batteries might be disposable or rechargeable. The compact and lightweight nature of lithium-ion batteries enhances the portability and inconspicuousness of insulin pumps [171]. While the majority of pumps include a wall adapter for charging, some may also provide USB charging for added convenience [172]. Low battery alerts are frequently present on pumps to prompt users to recharge the device prior to its complete depletion [173]. Patchable and non-patchable insulin pumps were categorized in Table 5 according to their power source.

Non-patchable insulin pumps generally depend on battery power as a result of their substantial dimensions and the requirement for a resilient motor or pumping mechanism to ensure efficient insulin delivery. These devices frequently operate on alkaline AA or AAA batteries, which offer dependable power for prolonged durations. On the other hand, rechargeable batteries are frequently incorporated into patchable pumps, which are more compact and convenient to transport. The significance of dependable power sources in mechanically powered pumps to guarantee precise insulin delivery is highlighted by the implementation of spring or push mechanisms. The Animas Vibe and IR 250 models, which rely on two packs of AA batteries, can provide continuous insulin administration for up to 3 weeks [174].

Insulin manufacturers specify that insulin temperatures should not exceed 37 °C. Therefore, temperature fluctuations in rechargeable insulin pumps may have significant implications for the stability of the insulin in the pump. By measurements, we have shown that lithium-ion rechargeable batteries, such as those currently used to power insulin pumps, showed substantial temperature increases during a full charging period in both ambient conditions and warm environments when compared with battery-operated pumps [57].

#### 3.2.6. Back Pressure

Insulin pump back pressure is predominantly caused by obstructions in the insulin infusion system (IIS). The progressive development of these occlusions may be caused by insulin fibrils obstructing the cannula outflow or tubing, cannula kinking, inflammation or hematoma-induced skin compression at the infusion site, or the displacement of the IIS. Certain insulin delivery devices, such as the Medtronic 670G, may increase insulin delivery rates in response to elevated glucose levels; this may cause back pressure concerns. Certain pumps, such as the Medtronic 670G, have the capability to escalate insulin delivery rates in response to elevated glucose levels, which may potentially worsen back pressure complications. In order to mitigate these concerns, patients may modify the parameters of the pump accordingly [175]. Furthermore, the existence of air pockets within the insulin infusion set (IIS) may also contribute to the development of back pressure, which may result in altered insulin delivery into the subcutaneous tissue or interruptions of insulin infusion [176]. Manufacturers often do not indicate back pressure as a critical characteristic since it might vary depending on the conditions particular to the pump and the patient.

#### 3.2.7. Size and Weight

Insulin pumps are designed to be lightweight and small wearables that improve user comfort and mobility. This requires designers to choose components based on size and power efficiency. Designers prioritize integrated solutions and small packaging, such as UCSP™ and wafer-level packaging (WLP), to satisfy needs [177]. Furthermore, progress in materials and design methodologies plays a significant role in the creation of more compact and lighter insulin pumps, hence improving the mobility and overall quality of life for individuals managing diabetes. The dimensions and weight of commercially available patchable and non-patchable insulin pumps are compared in Table 6.

The size and weight of the Niia Essential insulin pump, recently submitted for FDA approval, have not been disclosed. However, the pump is circular in shape and has a capacity to hold 300 units of insulin in its reservoir [208]. Another FDA-cleared pump, the Deka ACE pump, has not had its dimensions disclosed [209]. However, the MODD1 device by Modular Medical has a reservoir size that is equivalent to the Niia Essential device. The MODD1 device has just been submitted for FDA certification, but the particular details have not been revealed [210]. The Dibkit insulin pump, Sigi patch pump, and Unilife’s imperium wearable injector are currently undergoing development.

#### 3.2.8. Basal, Bolus, and Reservoir Size

Insulin pumps administer insulin in two main ways: by continuously infusing rapid-acting insulin throughout the day and night, which is called basal insulin delivery, and by providing individual doses of rapid-acting insulin that the user can take for meals or to correct high blood glucose levels, known as bolus dosing [211]. Insulin pumps commonly employ fast-acting insulin formulations such as insulin Lispro, Aspart, or Glulisine. Lispro and Aspart have been granted approval from the U.S. Food and Drug Administration (FDA) for use in insulin pump reservoirs for a maximum duration of 144 h. However, Glulisine necessitates replacement every 48 h, owing to the potential for crystallization [211]. The size of the insulin reservoir may limit the adoption of extended insulin pumps, as users with high total daily doses need to change reservoirs daily, while those with low total daily doses can last for seven days. To improve the usability of a seven-day insulin pump, a larger reservoir may be desirable, but space limitations in insulin pumps make this unlikely [36]. Therefore, reservoir sizes are determined to provide continuous insulin without affecting the insulin quality. Manufacturers take into account many aspects, such as the stability of insulin, and the likelihood of deterioration or crystallization, while deciding the size of the reservoir. By providing the optimum reservoir size, insulin pumps can deliver continuously over an extended period without compromising the quality. The two primary specifications, basal and bolus rate, of the insulin pumps along with their reservoir size are shown in Table 7.

#### 3.2.9. Age and Type of Diabetics

The study indicates that the effectiveness and safety of insulin pump therapy is largely equivalent in younger and elderly T1D patients. Basal/bolus ratios in CSII-treated patients were not significantly different between younger and older adult diabetic individuals. Patients over 50 years of age were willing to use advanced personal insulin pump options and tools such as dual wave/square bolus, Bolus Wizard, and continuous glucose monitoring just as frequently as younger T1D individuals [228]. Table 8 provides approved age groups and diabetes types for the specified insulin pumps.

While the Niia Essential patch pump has obtained FDA certification, it has not provided information regarding the age group or specific kind of diabetes for which it is intended. Currently, the age category and specific kind of diabetes patients for whom the Imperium patch pump, Dibkit insulin pump, and Osmotic WBI are being developed have not been determined. The age group and type of diabetes for which the non-patchable pumps Truecare, Insul, and YST-IVC are suitable have not been precisely specified.

#### 3.2.10. CGM, Types of Insulin, and Insertion Device

Insulin pumps frequently incorporate Continuous Glucose Monitors (CGMs) to offer real-time glucose monitoring data. In terms of insulin types, the majority of insulin pumps may be used with rapid-acting insulin, such as insulin Lispro, Aspart, or Glulisine. Insulin pumps are equipped with numerous types of insertion devices for placing infusion sets. These include manual insertion devices and automated insertion devices, that are mentioned in Table 9, each providing varying levels of user comfort and convenience.

#### 3.2.11. Patchable, Disposable, and Connection

Insulin pumps are equipped with different characteristics to improve their mobility, disposability, and connection in order to meet the varied requirements of patients who are controlling diabetes. Insulin pumps are usually compact and lightweight, making them easy to wear discreetly under clothes. This allows users to go about their everyday activities without any disruption. Certain pumps have disposable elements, including infusion sets and reservoirs, which streamline the management of insulin therapy by reducing the requirement for frequent changes of tubing and refills of reservoirs. Most of insulin pumps often utilize wireless connectivity through Bluetooth or radio frequency technologies to facilitate smooth connection with external devices like cell phones, continuous glucose monitors (CGMs), and insulin management apps. In Table 10, the connectivity and disposal characteristics of the patchable and non-patchable insulin pumps are detailed.

#### 3.2.12. Price and Wearing Time

Insulin pumps offer the best diabetes control, but their high cost makes them unaffordable to many. In the US, the cost of an insulin pump is around USD 6500, and the pump has a life expectancy of 3–4 years. Pump users must also purchase consumables to use with their devices. Insulin, infusion sets, pump cartridges, and filling syringes/needles cost between USD 2000 and USD 3000 per year. Those without health insurance or whose insurance only covers a percentage of these costs face a significant initial outlay and a continuing financial burden when opting for an insulin pump. In single-payer health systems, this high cost leads to the rationing of subsidized access [168].

The regular insulin pumps are approved for two to three days or with a planned set change at three days [64]. Some insulin pumps are designed/planned with a set change at seven days or more [65]. Table 11 displays the wearing time and cost of widely recognized insulin pumps available for purchase.

The costs of most commercially accessible pumps have been discovered on several web retailers. The cost of certain fuel pumps is contingent upon an individual’s health insurance coverage. To obtain the current pricing, it is advisable to contact the pump manufacturer and the health insurance provider, providing them with a valid prescription from a doctor.

## 4. Challenges and Future Directions

Although there are several advantages to CSII, the cost of these systems is much greater than MDI, which is a significant barrier to the widespread use of insulin pumps. Moreover, the complexity of micropump designs has been increasing over time, posing obstacles in guaranteeing their reliability. Recently, researchers have been developing new micropump designs to enhance insulin delivery. Among these advancements, magnetorheological-based micropumps are receiving considerable interest. Some designs use specialized one-way valves for a unidirectional pumping action, while others employ a series of electromagnets to force the fluid through the pump channel. For example, Stork first investigated the impact of electromagnets on fluid movement and developed an MRE peristaltic pump for fluid transportation [285]. Behrooz and Gordaninejad used a soft MRE membrane to study the microfluid transportation system by conveying Newtonian fluid [286,287]. Their work highlighted the significant impact of design parameters on performance. Ehsani and Nejat presented a simple conceptual design for a flexible-valve micropump that utilizes the magneto–fluid–structural interaction (MFSI) in three physics simulations [288]. Xufeng et al. proposed utilizing an MRE-based magneto-active pulse pump in three dimensions; however, their numerical analysis did not include the integration of valves in the microchannel [289]. Current micropump designs have specific drawbacks and limitations. For example, the initial Behrooz and Gordaninejad proposal shows a smaller pumping capacity. The micropump configuration that Ehsani and Nejat proposed exhibits a relatively slow response time when subjected to a magnetic field, in addition to a reduced actuation force. Their design faces major backflow issues due to the large space between the valve points and the upper wall. Similarly, there are response time delays and a reduced pumping capacity with the design proposed by Xufeng et al. Hence, there is still a need for improved magnetorheological pump designs that could function with faster response times, reduce backflow, and offer increased pumping capacity.

Due to the controlled properties under external magnetic fields, magnetorheological materials have attracted extensive attention [290,291,292,293]. Via the applied magnetic field, their rheological properties can be changed rapidly, continuously, and reversibly. In the domain of the architecture, automotive vehicles, and vibration controls, MR materials presently play important roles [290]. In general, there are two forms of magnetorheological materials; they are fluid [291,292,293,294,295], gel forms [296,297], and elastomers [298,299,300,301,302], which are widely applied in vibration absorbers [298,303], power transmission devices [302], magnetic actuated devices [304], and so on. Both anisotropic and isotropic magnetorheological elastomers (MREs) are prepared in the presence and absence of magnetic fields, respectively. For the improvement of MREs, different kinds as well as shapes of magnetic particles are used [305]. The magnetically induced body force or magnetic torques are used to control the deformation of MREs [306,307]. A gradient magnetic field can be used to achieve the magnetically induced body forces, although it is less energy efficient. The magnetic torque is always realized through a uniform magnetic field, which is generated by elaborated devices. For water conveying as well as the investigation of the influence of the scheduling of electromagnets preliminarily on the fluid transport, an MRE peristaltic pump is first proposed by Stark [308]. An MRE is a soft membrane, used to realize a microfluid transport system, and was applied by Behrooz and Gordaninejad [286] for conveying Newtonian fluid, and the performance is significantly affected by the design parameters, as also demonstrated by them. Using magneto–fluid–solid interaction (MFSI) simulation, which is based on MREs, Ehsani and Nejat [288] propose a simple flexible-valve micropump conceptual design. The mentioned MRE pumps have potential applications in implanting artificial organs in human bodies through utilizing simple mechanisms. Moreover, inside these micropumps, valves are mounted, which are only capable of conveying fluids unidirectionally and exhibiting intricate structures. A constant relative magnetic permeability is used to simplify the magnetic property of MREs [309,310,311,312]. 

One of the major issues faced by the existing technologies especially piezoelectric pumps is the clogging effect [313]. It occurs either inside the chamber or where the valve is located [314,315]. Clogging may happen when small particles are present in the fluid. It is also visible when bubble generates inside the chamber and gets trapped near the valve body due to cavitation. This decreases the overall performance of the insulin pump. Different approaches are being investigated to overcome this issue. Studies show that multiple trials are run to develop a self-cleaning pump, chamber modification, or an anti-clog valve design so that clogging effect can be addressed [316,317,318,319]. Overcoming this limitation will be another milestone for the insulin industry. 

Looking forward, there are several promising areas for advancing insulin pump technology, particularly in the realm of magnetorheological (MR) elastomer (MRE)-based pumps. Future research endeavors could prioritize enhancing the efficiency and response times of MRE-based pumps through innovations in material science and engineering techniques [320]. Moreover, integrating smart technologies, such as sensors and data analytics, alongside machine learning algorithms holds significant promise for optimizing insulin delivery in real-time [321]. This integration not only aims to improve treatment outcomes by adapting to individual patient needs, but also enhances the overall user experience by providing personalized and proactive diabetes management solutions [322].

Exploring novel materials beyond current MR elastomers and refining micropump designs are critical steps towards achieving more reliable and effective insulin delivery systems [323]. Advances in material science could lead to improvements in biocompatibility, durability, and performance metrics essential for long-term device reliability and patient safety.

Addressing these challenges and continuing to innovate will be pivotal for the future of diabetes management and the broader field of medical devices, ensuring that next-generation insulin pumps meet the evolving needs of healthcare providers and patients alike [324].

## 5. Conclusions

In conclusion, the comprehensive exploration of diabetes management presented in this report underscores the dynamic evolution of insulin delivery systems. The initial discussion delves into the multifaceted landscape of diabetes, covering types, risk factors, complications, and various treatment modalities. Emphasis is placed on the pivotal role of insulin and the nuanced understanding of its diverse physiological functions. The exploration extends to different insulin types, their characteristics, and therapeutic applications. The focus then shifts to the evolution of insulin delivery systems, showcasing remarkable advancements that have diversified options for individuals with diabetes. From traditional manual injections to sophisticated methods such as jet injectors, insulin pumps have played a significant role in enhancing patient convenience and precision in administration.

This paper specifically focuses on the recent advancements in patch pumps, highlighting their flexibility and patient-centric design. Key takeaways include the challenges in managing energy consumption, the diversity of patient needs addressed by patch pumps, and the innovative technological solutions employed. Practical challenges, such as tubing issues and wear-related changes, are acknowledged, with ongoing innovation aimed at enhancing user comfort and pump performance. The overview of patchable micropumps introduces various products with unique features and mechanisms. Ongoing technological advancements, including self-powered insulin patch pumps and smart insulin-delivery patches, demonstrate the continuous pursuit of precision and efficiency in glucose regulation.

This report also discusses alternative delivery methods, such as implantable and inhalable insulin pumps, along with the potential of nanotechnology for non-injectable insulin formulations. The challenges outlined include addressing specific user group needs, improving software and hardware for artificial pancreas systems, and enhancing the user-friendly features in patch pump platforms.

Our comparative study underscores the diverse landscape of insulin pumps and the continuous innovations aimed at providing tailored solutions for individuals with diabetes. The future holds the promise of even smaller, more efficient, and user-friendly devices, ultimately improving diabetes management and enhancing the quality of life for those affected by the condition.

## Figures and Tables

**Table 1:** Previous work on insulin pump.

Year	Author	Research Objectives	Ref.
2008	Tabakha et al.	Discussed various routes and very few conventional delivery systems.	[21]
2010	Jean-Louis Selam	Assessed insulin pen benefits, such asaccuracy, ease of use, patient satisfaction, quality of life, adherence. Analyzed cost savings potential with insulin pen adherence versus vial/syringe. Evaluated CSII cost-effectiveness versus MDI for type 1 diabetes. Investigated limitations of current insulin delivery devices on blood glucose control. Explored advancements in insulin administration, such as physiologic routes, artificial pancreas, and transplantation. Identified barriers to U.S. insulin pen adoption, proposed awareness strategies.	[22]
2011	Hovorka et al.	Discussed the artificial pancreas design, its components, clinical outcomes, pros and cons of automated closed-loop systems, and compared with conventional pump therapy.	[23]
2016	Shah et al.	Discussed different routes of insulin delivery.	[2]
2018	Easa et al.	Discussed and compared the available oral and non-evasive insulin delivery.	[24]
2019	Sora et al.	Discussed the pros and cons of CSII therapy, along with technical and clinical considerations for initiating patients on this treatment.	[25]
2019	Berget et al.	Provided an overview of insulin pump technologies, covering simple disposable pumps for type 2 diabetes to advanced automated systems. Discussed the clinical implications of these insulin pump technologies.	[26]
2021	Aiello et al.	Explored recent advancements (5 years), challenges, and opportunities in AID systems, particularly tailored for specific age groups and metabolic conditions.	[27]
2022	Sherr et al.	Highlighted AID system limitations, especially focusing on safety concerns.	
2022	Sabbagh et al.	Conducted a review on polymeric non-invasive insulin delivery and emphasized the uses of hydrogel as a promising insulin delivery system.	[28]
2022	Nallicheri et al.	Compared to a few AID systems only in US.	[29]
2022	Sugumar et al.	Explored non-invasive and patient-friendly insulin delivery routes, including chemical and physical enhancers.	[30]
2023	Bassi et al.	Discussed available AID systems in Europe for T1D patients, focusing on their effectiveness across different age groups.	[31]

**Table 2:** Roadmap for the specification comparison of the available insulin pump.

Table No	Description of the Table
Table 3	Manufacturer and release year of the available insulin pumps
Table 4	FDA approval of available insulin pumps and country of origin
Table 5	Insulin pumps and their powered technology with types
Table 6	Size and weight comparison of available insulin pumps
Table 7	Basal, bolus, and reservoir capacity comparison of available insulin pumps
Table 8	User’s age and type of diabetics for available insulin pumps
Table 9	CGM integration, types of insulin, and insertion devices for available pumps
Table 10	Portability, disposable item, and connection type of available insulin pump
Table 11	Wearing time and price of available insulin pump

**Table 3:** Manufacturer and release year of the available insulin pumps.

Pump Type	Pump Name	Manufacturer	Launching Year	Reference
Patchable	Omnipod 5	Insulet Corporation	2022	[87]
	Omnipod Dash	Insulet Corporation	2019	[88]
	EOPatch	EOFlow Inc, Now Medtronic	2022	[89]
	V-Go	Valeritas; acquired by Zeeland Pharma; acquired by Mankind Corporation	2011	[90,91]
	Cellnovo (Discont.)	Cellnovo	2014	[92]
	Accu Chek Solo	Roche	Initially made by Medingo and purchased by Roche in 2010	[93]
	SFC Fluidics	SFC Fluidics	Article published in 2019	[94]
	Niia essential	Pharmasens	After FDA approval; probably in 2024	[95]
	Equil Patch Pump	Microtech Medical	2017	[96]
	CeQur Simplicity (Previously One touch Via)	CeQur (Previously owned by J&J)	Commercialized by CeQur in 2021	[97]
	PAQ	CeQur	2012	[98]
	EasyPatch Pump	Medtrum	2016	[99]
	Touchcare Nano	Medtrum	2021	[100]
	Medisafe with	Terumo Corporation	2018	[101]
	Imperium Patch Pump	Unilife	2015	[102]
	Jewel Pump	Debiotech	2012	[66]
	Sigi	AMF Medical	2023	[103]
	Dibkit Insulin pump	IA Collaborative	-	[104]
	MODD1	Modular Medical	2023	[105]
	Osmotic WBI	Subcuject		[106]
	Kaleido	ViCentra	2023	[107]
Non-Patchable	Accu-Chek Spirit	Roche	2006	[108]
	T: Slim X2 with Basal IQ	Tandem Diabetes	2018	[109]
	T: Slim X2 with Control IQ	Tandem Diabetes	2018	[110]
	Tandem Mobi	Tandem Diabetes	2024	[111]
	Medtronic Paradigm	Medtronic	Paradigm 523: 2006; Paradigm 723: 2010	[112]
	MiniMed 630G	Medtronic	2016	[113]
	MiniMed 670G	Medtronic	2017	[114]
	MiniMed 770G	Medtronic	2020	[115]
	MiniMed 780G	Medtronic	2023	[116]
	iLet Bionic Pancreas	Beta Bionics	2023	[117]
	OneTouch Ping	Animas Corp.	2008	[118]
	Animas Vibe	Animas Corp.	2011	[119]
	IR-1250	Animas Corp.	2005	[120]
	Dana II	Sooli	2000	[121]
	Nipro Amigo	Nipro	2001	[122,123]
	Deltec Cozmo	Smith’s Medical	2002	[124]
	Mylife Ypso Pump	Ypsomed AG	2016	[125]
	Truecare	Apex Medical	-	[126]
	YST-IVC Insulin Pump	Shanghai Umitai Medical Technology Co., Ltd.	-	[127]
	Insul	Agva	-	[128]
	Deka ACE Pump	DEKA Research & Development Corp	2023	[129]
	Twiist	Sequel Med Tech	2024	[130]

**Table 4:** FDA approval of available insulin pumps and country of origin.

Pump Type	Pump Name	FDA Approval	Available Countries	Reference
Patchable	Omnipod 5	FDA	USA, UK, and Germany	[131]
	Omnipod Dash	FDA	Sweden, Finland, Norway, Denmark, France, Belgium, Germany, Austria, Switzerland, Israel, USA, UK, The Netherlands, and Italy.	[132]
	EOPatch	CE	South Korea	[133]
	V-Go	FDA-2010-Humalog FDA-2011-Novolog FDA-2012-Type 2 CE	USA, EU, and Australia	[134,135]
	Cellnovo (Discontinued)	CE Mark	France, UK, The Netherlands, Italy, Spain, Australia, New Zealand, Israel, Greece, and Cyprus	[136,137]
	Accu Chek Solo	FDA	US, UK, Australia, Poland, Switzerland	[138,139]
	Niia essential	ISO 13,485	Switzerland	[95]
	Equil Patch Pump	CE	Asia Pacific, Europe, Middle East, Africa, and Latin America	[96]
	CeQur Simplicity (Previously One touch Via)	FDA, CE Mark	Europe, USA	[140]
	PAQ	CE Mark	Europe	[141]
	EasyPatch Pump	CE	Limited usage in Europe	[142]
	Touchcare Nano	CE Mark	The Netherlands, UK, France, Germany, Denmark, Finland, and Sweden	[143,144]
	Medisafe with	CE Mark	Japan	[145]
	MODD1	-	Europe	[146]
	Kaleido	-	The Netherlands France, Germany	[147,148]
Non-Patchable	Accu-Chek Spirit	FDA	Selected countries in the Middle East and Africa region	[149,150]
	T: Slim X2 with Basal IQ	FDA	USA, Canada	[109,151]
	T: Slim X2 with Control IQ	FDA	USA, Canada	[110,152]
	Tandem Mobi	FDA	USA	[111]
	Medtronic Paradigm	FDA	USA	[112]
	MiniMed 630G	FDA	USA	[113]
	MiniMed 670G	FDA	USA, Europe	[114]
	MiniMed 770G	FDA	USA	[115]
	MiniMed 780G	FDA, CE	USA, Europe	[153,154]
	iLet Bionic Pancreas	FDA	USA, UK	[155,156]
	OneTouch Ping	FDA	USA, Europe, Australia, New Zealand, and Canada	[157]
	Animas Vibe	FDA	USA, Europe, Australia, New Zealand, and Canada	[158]
	IR-1250	FDA	USA	[159]
	Dana II	FDA	USA	[121]
	Nipro Amigo	FDA	USA	[160]
	Deltec Cozmo	FDA	Worldwide	[161,162]
	Mylife Ypso Pump	CE	Australia, Canada, and Europe	[163,164]
	Truecare	CE	Europe, India, China	[126]
	YST-IVC Insulin Pump	-	China	[127]
	Insul	-	India	[128]
	Deka ACE Pump	Yes	USA	[129]
	Twiist	FDA	-	[130]

**Table 5:** Insulin pumps and their powered technology with types.

Powered Technology	Pump Type	Insulin Pumps
Battery powered	Patch pump	Omnipod 5, Omnipod Dash, EO Patch, Accu-Check Solo, SFC Fluidics, Niaa essential, Equil patch pump, Touchcare nano, Medisafe, Jewel pump, Symboize, Sigi, MODD1,
	Non-patch pump	MiniMed 670G, 770G, 780G, ilet Bionic Pancreas, One touch ping, Animas vibe, IR-250, Dana, Nipro amigo, Deltec cosmo, Mylife Ypso, Truecare, YST-IVC, Insul, Deka Ace pump
Mechanically powered	Patch pump	V-Go, CeQur Simplicity PAQ, Libertas, Osmotic WBI
	Non-patch pump	-

**Table 6:** Size and weight comparison of available insulin pumps.

Pump Type	Pump Name	Size	Weight	Ref.
Patchable	Omnipod 5	39 × 52 × 14.5 mm (Pod size)	28.35 g with an empty reservoir.	[178]
	Omnipod Dash	39 × 52 × 14.5 mm (Pod size)	28.35 g with an empty reservoir.	[179]
	EOPatch	49.5 × 39 × 14.5 mm	29.4 g (Wearable unit weight: 26 g/excluding insulin)	[180]
	V-Go	60.96 × 33.02 × 12.7 mm.	19.8 gm to 51.03 gm	[181]
	Cellnovo (Discontinued)	50.8 × 38.1 × 15.24 mm	-	[66]
	Accu Chek Solo	61 × 38 × 13 mm	29 g	[169]
	SFC Fluidics Patch Pumps	55 × 58 × 10 mm	113.4 g	[94,182]
	Equil Patch Pump	59.5 × 40.0 × 12.1 mm	23 g	[183]
	CeQur Simplicity (Previously One touch Via)	63 × 35 × 8 mm	10 g	[184]
	PAQ	60 × 77 × 18 mm	36 g	[185]
	Easypatch Pump	40.5 × 31.5 × 11.5 mm	13.8	[186]
	Touchcare Nano	40.5 × 31.5 × 11.5 mm	13.8 g	[187]
	Medisafe with	77.9 × 40.1 × 18.9 mm	34 g	[145,188]
	Jewel Pump	70 × 40 × 13 mm	25 g	[189]
	Osmotic WBI	84 × 48 × 18.5 mm	-	[190]
	Kaleido	50 × 35 × 12.5 mm	19 g	[147]
Non-Patchable	Accu-Chek Spirit	82.5 × 56 × 21 mm	Empty approx. 80 g; insulin pump with battery, full plastic cartridge, and infusion set; approx. 110 g	[150]
	T: Slim X2 with Basal IQ	79.5 × 50.8 × 15.24 mm	111.98 g	[191]
	T: Slim X2 with Control IQ	79.5 × 50.8 × 15.24 mm	111.98 g	[192]
	Tandem Mobi	37.85 × 45.72 × 12.7 mm	102.058 g	[193]
	Medtronic Paradigm	523: 51 × 83 × 20 mm 723: 51 × 94 × 21 mm	523: 95 g 723: 102 g	[194]
	MiniMed 630G	97 × 53.3 × 24.3 mm	104.893 g	[195]
	MiniMed 670G	96.8 × 53.6 × 24.9 mm	106 g	[196]
	MiniMed 770G	97 × 53.3 × 24.3 mm	106 g	[197]
	MiniMed 780G	97 × 53.3 × 24.3 mm	106 g	[198]
	iLet Bionic Pancreas	91 × 50 × 15 mm	110 g	[199]
	OneTouch Ping	97 × 62 × 28 mm	110.5 g	[200]
	Animas Vibe	83 × 51 × 22 mm	105 g	[201]
	IR-1250	77 × 51 × 18 mm	87.88 g	[202]
	Dana II	91 × 45.5 × 20 mm	53 g	[203]
	Nipro Amigo	83.312 × 55.372 × 23.622 mm	87.88 g	[204]
	Deltec Cozmo	81.28 × 45.72 × 22.86 mm	77 g	[204]
	Mylife Ypso Pump	78 × 46 × 16 mm	83 g	[164]
	Truecare	88 × 58 × 20 mm	105 g	[205,206]
	YST-IVC Insulin Pump	92 × 52 × 20 mm	55 g	[127]
	Insul	113 × 67 × 24 mm	250 g	[128]
	Twiist	50.8 × 50.8 mm	-	[207]

**Table 7:** Basal, bolus, and reservoir capacity comparison of available insulin pumps.

Pump Type	Pump Name	Basal	Bolus Rate	Reservoir Capacity	Ref
Patchable	Omnipod 5	0 to 30 units per hour in 0.05-unit	0.05 to 30 units. Increments of 0.05 units	200 Unit. Can provide up to 72 h of continuous insulin delivery	[178]
	Omnipod Dash	From 0 to 30 units per hour in 0.05-unit increments	From 0.05 to 30 units. Increments of 0.05 units.	200-Unit reservoir built into the pod	[179]
	EOPatch	Flexible basal	Flexible	Min. 80 Unit~Max. 200 Unit	[180]
	V-Go	20, 30 or 40 units per 24 h	36 units per 24 h in 2 unit	V-Go 20: 56 Unit total V-Go 30: 66 Unit total V-Go 40: 76 Unit total	[212]
	Cellnovo (Discontinued)	0.05–5 U/h	0.05–30 units.	150 Unit	[213]
	Accu Chek Solo	0.1 U up to under 5.0 U: increments of 0.01 units 5.0 U up to under 25.0 U: increments of 0.1 units	Minimum: 0.2 units Maximum: 50 units	80–200 Unit	[169]
	SFC Fluidics Patch Pumps	0.5 U/h	2 units	300 Unit	[94,182]
	Niia essential	-	-	300 Unit	[214]
	Equil Patch Pump	min: 0.025 U/h/max: 35 U/h	min: 0.025 U/max: 35 U	200 Unit	[183]
	CeQur Simplicity (Previously One touch Via)	Only Bolus	Bolus only 2-unit dose/Click	200 Unit	[215,216]
	PAQ	16, 20, 24, 32, 40, 50, or 60 units per day	2 units	330 Unit	[66]
	EasyPatch Pump	0–0.05 U/h	22.35 U	200 Units	[217]
	Touchcare Nano	25 U/h	Minimum bolus: 0.05 Units (0.05 U/2 secs)	200 Unit	[187]
	Medisafe with	0.05 U/h (0.00 to 35.00 U/h)	0.1 U (0.1 to 25.0 U) Normal bolus (speed: 1.5 U/min) Quick bolus (speed: 15 U/min)	200 Unit	[188]
	Imperium Patch Pump	-	-	500 Unit	[102]
	Jewel Pump	-	-	500 Unit	[189]
	Sigi	0.025 U	0.2 U	160 Unit	[218]
	MODD1	-	-	300 Unit	[219]
	Osmotic WBI	-	-	300 Unit	[220]
	Kaleido	0.05 U/h -/5 U/h	0.05 U–30 U	200 Unit	[221]
Non-Patchable	Accu-Chek Spirit	Min. = 0.05 U/h (U/h), Max. = 50 U/h. There are 24 hourly basal rates, adjustable in unit increments of 0.01 (up to 1.00 U/h), 0.05 (up to 10.0 U/h), and 0.1 (up to 50.0 U/h).	Max 50 insulin units; Quick bolus, which is adjustable in unit increments of 0.1, 0.2, 0.5, 1.0, and 2.0; Standard bolus, extended Bolus, and Multiwave Bolus are adjustable in fixed increments of 0.1 units; Duration of extended bolus and multiwave bolus are adjustable in intervals of 15 min (from 15 min up to 12 h).	315 Unit/Accu-chek Spirit 3.15 mL cartridge system and Accu-chek 3.15 mL plastic cartridge with a lure-lock connection;	[222]
	T: Slim X2 with Basal IQ	From 0.1 to 15 units per hour in 0.001-unit increments	From 0.05 to 25 units in 0.01-unit increments with an option for up to an additional 25 units	300 Unit	[191]
	T: Slim X2 with Control IQ	From 0.1 to 15 units per hour in 0.001-unit increments	From 0.05 to 25 units in 0.01-unit increments with an option for up to an additional 25 units	300 Unit	[192]
	Tandem Mobi	0.1–15	0.05 to 25; Bolus increment 0.01	200 Unit	[193]
	Medtronic Paradigm	0.025–35 U/h	0.025 units for bolus amounts in the range of 0.025 to 0.975 units 0.05 units for bolus amounts larger than 0.975 units	523: 176 Unit 723: 300 Unit	[194]
	MiniMed 630G	From 0.025 to 35 units per hour in 0.025-unit increments for rates of 0.025–0.975 units/hr and increments of 0.05 units for rates of 1.00–9.95 U/h	Bolus Range: From 0.025 to 25 units. Increments of 0.025 units.	300 Unit	[195]
	MiniMed 670G	• 0.025 U/h for basal amounts in the range 0 to 0.975 U • 0.05 U/h for basal amounts in the range 1 to 9.95 U • 0.1 U/h for basal amounts of 10 to 35 U	Bolus Range: From 0 to 25 units. Increments of 0.025/0.05/0.10 units.	300 Unit	[196]
	MiniMed 770G	• 0.025 U/h for basal amounts in the range 0 to 0.975 U • 0.05 U/h for basal amounts in the range 1 to 9.95 U • 0.1 U/h for basal amounts of 10 to 35 U	Bolus Range: From 0 to 25 units. Increments of 0.025/0.05/0.10 units.	300 Unit	[197]
	MiniMed 780G	• 0.025 U/h for basal amounts in the range 0 to 0.975 U • 0.05 U/h for basal amounts in the range 1 to 9.95 U • 0.1 U/h for basal amounts of 10 to 35 U	Bolus Range: From 0 to 25 units. Increments of 0.025/0.05/0.10 units.	300 Unit	[198]
	iLet Bionic Pancreas	±5% Max (10 U/h) and Intermediate (1.0 U/h)/±15% Min Basal (0.1 U/h)	±5% Max (30 U)/Intermediate (5 U)/Min (0.5 U)	180 Unit	[223]
	OneTouch Ping	0.025–25 U/h in 0.025 U/h steps	0.05–35 U in 0.05 U steps	200 Unit	[200]
	Animas Vibe	0.025 U/h across all available ranges (0.025 U/h to 25.00 U/h)	Bolus increment of 0.05 U across all available bolus ranges (0.05 U to 35.00 U)	200 Unit	[158]
	IR-1250	0.025–25 U/h in 0.025 U/h steps	0.05–35 U in 0.05 U steps	200 Unit	[224]
	Dana II	0.04 U/h	0 to 80 units	300 unit	[203]
	Nipro Amigo	0.05 U–30 U/h	0.05 Units	300 Unit	[204,225]
	Deltec Cozmo	0.05–35 U/h	0.05, 0.1 visual; 0.05, 0.1, 0.5, 1.0 visual or audio	300 Unit	[226]
	Mylife Ypso Pump	0.1 U to 30.0 U	0.1 U, 0.5 U, 1.0 U and 2.0 U	160 Unit	[164]
	Truecare	0.1–35 U/h	0.1–25 U	300 Unit	[205]
	YST-IVC Insulin Pump	0.05 U/h	N/A	300 Unit	[127]
	Insul		Up to 0.025 U/h	500 Units	[128]
	Deka ACE Pump	0–30 U/h	0.05–25, 0.01-unit increments	180 Unit	[227]
	Twiist	-	-	300 Unit	[130]

**Table 8:** User’s age and type of diabetics for available insulin pumps.

Pump Type	Pump Name	Age Limit	Types of Diabetics	Ref.
Patchable	Omnipod 5	2 years and older	Type 1	[178]
	Omnipod Dash	Cleared for all ages.	Type 2	[179,229]
	EOPatch	-	Type 1	[230]
	V-Go	21 years and older	Type 1 and 2	[231]
	Cellnovo (Discontinued)	Targeted younger T1DM including all other age groups	Type1	[213]
	Accu Chek Solo	2 years and older	Type 1	[139]
	SFC Fluidics Patch Pumps	-	Type 1 and 2	[232]
	Equil Patch Pump	18 years and older	Type 1 and 2	[183,233]
	CeQur Simplicity (Previously One touch Via)	21 years and older	Type 1 and 2	[215,234]
	PAQ	-	Type 2	[185]
	EasyPatch Pump	-	Type 1	[99]
	Touchcare Nano	-	Type 2	[144]
	Medisafe with	18 years and older	Type 1	[235]
	Jewel Pump	-	Type 1	[189]
	Sigi	18 years and older	Type 1	[236]
	Kaleido	18 years and older	Type 1	[221]
Non-Patchable	Accu-Chek Spirit	-	Type 1 and 2	[149]
	T: Slim X2 with Basal IQ	6 years and older	Type 1	[237]
	T: Slim X2 with Control IQ	6 years and older	Type 1	[237]
	Tandem Mobi	6 years and older	Type 1	[111]
	Medtronic Paradigm	7 years and older	Type 1	[238]
	MiniMed 630G	16 years and older	Type 1	[113]
	MiniMed 670G	7 years and older	Type 1	[114,239]
	MiniMed 770G	2 years and older	Type 1	[115]
	MiniMed 780G	7 years and older	Type 1	[198]
	iLet Bionic Pancreas	6 years and older	Type 1	[156]
	OneTouch Ping	18 years and older	Type 1	[240,241]
	Animas Vibe	2 years and older	Type 1	[158,240]
	IR-1250	-	Type 1	[224]
	Dana II	7 years and older	Type 1	[242]
	Nipro Amigo	-	Type 1 and 2	[123]
	Deltec Cozmo	9 years to 76 years	Type 1	[243,244]
	Mylife Ypso Pump	1 years and older	Type 1	[245]
	Deka ACE Pump	13 years and older	Type 1	[129]
	Twiist	6 years and older	Type 1	[130]

**Table 9:** CGM integration, types of insulin, and insertion devices for available pumps.

Pump Type	Pump Name	CGM Integration	Types of Insulin Used	Insertion Device	Ref.
Patchable	Omnipod 5	Dexcom CG6	Novolog/NovoRapid, Humalog, Fiasp, Admelog, Lyumjev, or Apidra.	No	[246]
	Omnipod Dash	Contour Next One blood glucose meter (no integration with BGM, only connected with PDM)	Novolog/NovoRapid, Humalog, Fiasp, Admelog, Lyumjev, or Apidra.	No	[179]
	EOPatch	CGM technology from Medtronic, Meal Detection Technology™ algorithm [Link]	Humalog	No	[230]
	V-Go	No	U-100 insulin	Comes with a built-in 30-gauge, 4.6-mm stainless-steel needle with a 90-degree insertion angle. The needle retracts into the device after use to reduce the risk of sharps injury.	[181,212]
	Cellnovo (Discontinued)	Touch screen, app-based handset, built in BGM	-	Yes	[213]
	Accu Chek Solo	No	U-100 insulin	Yes	[247]
	SFC Fluidics Patch Pumps	-	Dual Hormone	-	[248]
	Niia essential	Yes (Integrated and external)	-	Yes	[214]
	Equil Patch Pump	Integrated	U-100 insulin	Yes	[183]
	CeQur Simplicity (Previously One touch Via)	No CGM	U-100 insulin	Yes	[215]
	PAQ	No CGM	U-100 insulin	Yes	[185]
	EasyPatch Pump	S6 EasySense	U-100 insulin	No	[217]
	Touchcare Nano	Touch care nano CGM	U-100 insulin	No	[187]
	Medisafe with	Yes	-	Needs other devices, as well as a strap and infusion set	[188]
	Imperium Patch Pump	No	-	No	[249]
	Jewel Pump	No	-	-	[250]
	Sigi		U-100 insulin	Yes	[218]
	Dibkit Insulin pump	No	-	-	[104]
	MODD1	-	-	Yes	[219]
	Osmotic WBI	No	-	-	[220]
	Kaleido	Yes	U-100 insulin	Yes	[251]
Non-Patchable	Accu-Chek Spirit	No	U-100 insulin	Yes	[252]
	T: Slim X2 with Basal IQ	Dexcom G6 or Abbott Libre 2 ICGM	U-100 insulin	No	[253]
	T: Slim X2 with Control IQ	Dexcom G6, G7, and Libre 2 Plus	U-100 insulin	No	[192,254]
	Tandem Mobi	Dexcom G6	U-100 insulin	No	[193]
	Medtronic Paradigm	Yes	U-100 insulin	No	[194]
	MiniMed 630G	Guardian 3 CGM, PID	U-100 insulin	No	[255]
	MiniMed 670G	Guardian 3 CGM, PID	U-100 insulin	No	[196]
	MiniMed 770G	Guardian Sensor 3/Zeus CGM (MPC Algorithm)	U-100 insulin	No	[197]
	MiniMed 780G	Guardian Sensor 3/Synergy disposable CGM (DreaMed Advisor Pro fuzzy logic algorithm)	U-100 insulin	No	[198]
	iLet Bionic Pancreas	Dexcom G6 or G7 Senseonics CGM, Gen 4 PDA, PID Algorithm	U-100 insulin	No	[223]
	OneTouch Ping	No	U-100 insulin	No	[256]
	Animas Vibe	Dexcom G4	U-100 insulin	No	[201,256]
	IR-1250	No	U-100 insulin	No	[224]
	Dana II	No	U-100 insulin	Yes	[203]
	Nipro Amigo	No	U-100 insulin		[123]
	Deltec Cozmo	No	U-100 insulin	No	[124]
	Mylife Ypso Pump	Yes	U-100 insulin	Yes	[164]
	Truecare	No	U-100 insulin	No	[205]
	YST-IVC Insulin Pump	No	-	No	[127]
	Insul	No	-	Yes	[128]
	Deka ACE Pump	Yes	U-100 insulin	Yes	[227]
	Twiist	Yes	U-100 insulin	-	[130]

**Table 10:** Portability, disposable item, and connection type of available insulin pumps.

Pump Type	Pump Name	Disposable	Connection	Ref.
Patchable	Omnipod 5	Yes	Wireless	[257]
	Omnipod Dash	Yes	Wireless	[178]
	EOPatch	Yes	Wireless	[258]
	V-Go	Yes	No connection	[259]
	Cellnovo (Discontinued)	Cartridge is disposable	Wireless	[213]
	Accu Chek Solo	Disposable adhesive pump holder, disposable adhesive infusion cannula, disposable 200-unit reservoir,	Wireless	[247]
	SFC Fluidics Patch Pumps	Yes	Wireless	[94]
	Niia essential	Yes	Wireless	[214]
	Equil Patch Pump	Yes	Wireless	[260]
	CeQur Simplicity (Previously One touch Via)	Yes	Wireless	[261]
	PAQ	Yes	-	[185]
	EasyPatch Pump	Yes	Wireless	[99]
	Touchcare Nano	Yes	Wireless	[187]
	Medisafe with	Insulin reservoir	Wireless	[188]
	Imperium Patch Pump	Yes	No	[102]
	Jewel Pump	Yes	Wireless	[262]
	Sigi	Yes	Wireless	[218]
	Dibkit Insulin pump		Wireless	[104]
	MODD1	Yes	Wireless	[105]
	Osmotic WBI	Yes	Wireless	[220]
	Kaleido	Yes	Wireless	[251]
Non-Patchable	Accu-Chek Spirit	Yes	Wireless	[150,263]
	T: Slim X2 with Basal IQ	Yes	Wireless	[253]
	T: Slim X2 with Control IQ	Yes	Wireless	[254]
	Tandem Mobi	Disposable cartridge	Wireless	[264]
	Medtronic Paradigm	Disposable reservoir and infusion set	Wireless	[194]
	MiniMed 630G	Disposable reservoir and infusion set	Wireless	[255]
	MiniMed 670G	Disposable reservoir and infusion set	Wireless	[196]
	MiniMed 770G	Disposable reservoir and infusion set	Wireless	[197]
	MiniMed 780G	Disposable reservoir and infusion set	Wireless	[198]
	iLet Bionic Pancreas	Yes	Wireless	[223]
	OneTouch Ping	Disposable Insulin Cartridge	Wireless	[200]
	Animas Vibe	Disposable Insulin Cartridge	Wireless	[201]
	IR-1250	Disposable Insulin Cartridge	Wireless	[224]
	Nipro Amigo	-	No	[122]
	Dana II	Yes	-	[203]
	Deltec Cozmo	Yes	-	[265]
	Mylife Ypso Pump	Yes	Wireless	[266]
	Truecare	-	No	[205]
	YST-IVC Insulin Pump	-	No	[127]
	Insul	Yes	Wireless	[128]
	Deka ACE Pump	Yes	Wireless	[129]
	Twiist	Yes	Wireless	[130]

**Table 11:** Wearing time and price of available insulin pump.

Pump Type	Pump Name	Wearing Time	Price (USD)	Ref.
Patchable	Omnipod 5	3 days	600–700	[267,268]
	Omnipod Dash	3 days	600–700	[269,270]
	EOPatch	EOPatch can provide up to 3.5 days (84 h) of continuous insulin delivery, which allows for twice-a-week compliance, improving user compliance. The device can also withstand water for 24 h	-	[258]
	V-Go	24 h	530	[259,271]
	Cellnovo (Discontinued)	3 days	-	[213]
	Accu Chek Solo	3 days	-	[247]
	SFC Fluidics	3 days	-	[182]
	Niia essential	3 days	-	[272]
	Equil Patch Pump	2–3 days	4100	[183,273]
	CeQur Simplicity (Previously One touch Via)	3 days	450	[215,274]
	PAQ	3 days	-	[185]
	EasyPatch Pump	3 days	-	[142]
	TouchCare Nano	3 days	595	[275]
	Imperium Patch Pump	Multiday	-	[102]
	Jewel Pump	6 days	6500	[262,276]
	MODD1	3 days	-	[219]
	Kaleido	3 days	-	[251]
Non-Patchable	Accu-Chek Spirit		516	[277]
	T: Slim X2 with Basal IQ	2–3 days	4000	[253,278]
	T: Slim X2 with Control IQ	2–3 days	4000	[253,279]
	Tandem Mobi	2–3 days	8000	[193,264]
	MiniMed 630G	-	8000	[280]
	MiniMed 670G	-	8000	[239]
	MiniMed 770G	-	7250	[281]
	MiniMed 780G	-	7250	[282]
	Animas Vibe	7 days	7150	[158,283]
	Mylife Ypso Pump	-	6400	[284]
	Insul	-	300	[128]
	Deka ACE Pump	3 days	-	[227]
